# Genetically similar temperate phages form coalitions with their shared host that lead to niche-specific fitness effects

**DOI:** 10.1038/s41396-020-0637-z

**Published:** 2020-04-02

**Authors:** Jonelle T. R. Basso, Nana Y. D. Ankrah, Matthew J. Tuttle, Alex S. Grossman, Ruth-Anne Sandaa, Alison Buchan

**Affiliations:** 1 Department of Microbiology, University of Tennessee Knoxville, 1311 Cumberland Avenue, 307 Ken and Blaire Mossman Bldg., Knoxville, TN 37996 USA; 2 Department of Biological Sciences, University of Bergen, PO 7803, N-5020 Bergen, Norway; 3 Present Address: Department of Entomology, Cornell University, 5136 Comstock Hall, Ithaca, NY 14853 USA

**Keywords:** Bacteriophages, Microbial ecology

## Abstract

Temperate phages engage in long-term associations with their hosts that may lead to mutually beneficial interactions, of which the full extent is presently unknown. Here, we describe an environmentally relevant model system with a single host, a species of the Roseobacter clade of marine bacteria, and two genetically similar phages (ɸ-A and ɸ-D). Superinfection of a ɸ-D lysogenized strain (CB-D) with ɸ-A particles resulted in a lytic infection, prophage induction, and conversion of a subset of the host population, leading to isolation of a newly ɸ-A lysogenized strain (CB-A). Phenotypic differences, predicted to result from divergent lysogenic-lytic switch mechanisms, are evident between these lysogens, with CB-A displaying a higher incidence of spontaneous induction. Doubling times of CB-D and CB-A in liquid culture are 75 and 100 min, respectively. As cell cultures enter stationary phase, CB-A viable counts are half of CB-D. Consistent with prior evidence that cell lysis enhances biofilm formation, CB-A produces twice as much biofilm biomass as CB-D. As strains are susceptible to infection by the opposing phage type, co-culture competitions were performed to test fitness effects. When grown planktonically, CB-A outcompeted CB-D three to one. Yet, during biofilm growth, CB-D outcompeted CB-A three to one. These results suggest that genetically similar phages can have divergent influence on the competitiveness of their shared hosts in distinct environmental niches, possibly due to a complex form of phage-mediated allelopathy. These findings have implications for enhanced understanding of the eco-evolutionary dynamics of host-phage interactions that are pervasive in all ecosystems.

## Introduction

Temperate phages may engage in long-term association with their bacterial hosts that can lead to mutually beneficial interactions. It is well established that prophages can offer their hosts benefits, including resistance to superinfection by homologous phages [1–4] and enhanced virulence through prophage-encoded toxins [e.g., 1, 5, 6]. However, the roles of prophages in enhancing both host and phage fitness are broadening in scope and complexity [5, 7].

Prophages have frequently been referred to as “time bombs” [e.g., 3], in which the nature of the host-phage relationship hinges upon the physiological status of the host. The most commonly cited trigger of prophage induction is damage of the host’s DNA, typically the result of extrinsic factors such as UV radiation or chemical toxins, which induces a molecular cascade of events culminating in expression of prophage-encoded lytic genes [5, 8]. However, intrinsic factors may also promote activation of the lytic life cycle, a process termed spontaneous prophage induction (SPI) [6]. Even under seemingly optimal cultivation conditions, SPI occurs with low frequency in populations [range 0.09–3.1%; 9–13]. SPI is often considered a detrimental process for the host as a fraction of cells is continuously lost by phage-mediated cell lysis. Yet, benefits of SPI on bacterial fitness have recently been recognized, including the release of extracellular DNA, which can be important for biofilm formation [6] and production of phages as weapons in competition with susceptible hosts [14].

Lysogeny is hypothesized to be prevalent in marine environments [15, 16], where approximately half of sequenced bacterial genomes contain prophage [3, 7]. The abundance of marine temperate viruses is further supported by culture-independent approaches [17, 18]. Quantitative estimates of the prevalence of lysogeny in the ocean are principally derived from field-based mitomycin C induction experiments and vary widely, ranging from 0 to 71% [19–21]. This observed variation is predicted to reflect environmental conditions (e.g., host productivity and abundance) that drive temperate phages into either a lysogenic or lytic state [19, 22–26] and has been the focus of recent debate (e.g., [27–29]). In contrast, a role for spontaneous induction has not been broadly considered in a marine context.

Roseobacters are abundant members of microbial assemblages in both planktonic and surface-associated marine niches [30–32] and prophages are common in genomes of cultured representatives [33, 34]. Thus, this environmentally relevant group of heterotrophic marine bacteria presents an opportunity to study host-phage interactions in the context of lysogeny. *Sulfitobacter* sp. strain CB2047 and its infecting temperate phage ɸ-A were originally isolated from a phytoplankton bloom [35, 36]. Genome sequence analyses of the host revealed it was lysogenized with a prophage, denoted ɸ-D, that shares a high degree (79%) of nucleotide identity with ɸ-A [36]. Here, we report the complex interactions of this two-phage shared-host system.

## Methods

### Bacterial growth, prophage induction, and infection

*Sulfitobacter* sp. strain CB2047 (henceforth CB-D) was originally isolated from an *Emiliania huxleyi* phytoplankton bloom in Raunefjorden, Norway [36]. CB-D, and its derivative CB-A, were routinely grown at 25 °C in the dark at 200 rpm on Standard Marine Media (SMM), an artificial sea water medium supplemented with 0.11% yeast extract and 0.2% tryptone [37]. Phages were propagated by induction of exponentially growing lysogenic cultures with mitomycin C (0.5 µg/ml) following standard approaches [38]. Superinfections were performed by phage addition to early exponential phase cultures of permissive hosts (OD_540nm_ = 0.17) at a multiplicity of infection of 0.06. These infections yield mixed phage populations due to concurrent induction of the resident prophage.

### Phage enumeration

Phage abundance was monitored using plaque assay and quantitative PCR (qPCR). Plaque assays were performed on SMM using standard approaches [39]. qPCR assays used unique phage-specific primers (Table S1) and were performed with a DNA Engine Opticon 2 system with the Opticon Monitor 3.1.32 software package (Bio-Rad Laboratories, Inc., Hercules, CA). qPCRs were in 25 µL reactions with 12.5 µL SYBR Premix Ex Taq cocktail RR041 (Perfect Real Time; Takara Bio, Inc., Shiga, Japan), 500 nM primers, and 10 μl of phage DNA. Thermocycling conditions were as follows: 95 °C for 2 min, 40 cycles of 95 °C for 20 s, 57 °C for 20 s, 72 °C for 20 s, followed by 72 °C for 5 min. Melt curves consistently showed single peaks per primer set, indicating high specificity. Standards were developed from plasmids containing cloned sequences and standard curves (correlation between log of gene copy numbers and Ct) devised. Correlation coefficients for all standard curves were ≥ 0.99.

### Gene expression assays

Gene expression was quantified using quantitative reverse transcription-PCR (qRT-PCR) assays. Nucleic acids were extracted using AllPrep DNA/RNA Mini Kits (Qiagen, Valencia, CA) following manufacturer’s instructions. After extraction of RNA, DNA was removed using the TURBO DNA-free Kit (Ambion, Austin, TX). The resulting RNA samples were converted to cDNA using M-MLV Reverse Transcriptase and random hexamers (Invitrogen, Carlsbad, CA) following manufacturer’s instructions. M-MLV RT was heat inactivated by 15 min at 70 °C. qPCR was performed as described above.

Transcripts diagnostics of the host SOS response, phage DNA replication/repair, phage excision/cell lysis and prophage integration were quantified and normalized to the expression of three host reference genes (*alaS*, *map*, and *rpoC*) selected using previously described criteria [40]. Primers are shown in Table S1.

### Genome analysis

Genomic DNA was isolated for both CB-A and CB-D using standard phenol/chloroform extraction procedures [41] and sequenced with the Illumina HiSeq platform at the Genomic Services Lab (HudsonAlpha Institute for Biotechnology, Huntsville, AL). *Sulfitobacter* sp. strain CB-D was re-sequenced to confirm the original sequence [36]. Genome reads were assembled using CLC Genomics Workbench version 7.5.1 (QIAGEN). Reads were independently mapped to the original CB-D genome sequence (JPOY00000000) using Map Reads to Reference, followed by Local Sequence Realignment. Average read coverage was 278 for CB-D and 292 for CB-A. The Fixed Ploidy Variant Detection tool was used to identify nucleotide differences between assembled genome contigs.

### Biofilm assays

Clear flat-bottomed 96-well polypropylene plates (co-culture experiments) or 5 ml polypropylene tubes (monoculture experiments) were inoculated with 100 µl and 1 ml of overnight cultures, respectively, grown in SMM (diluted in fresh medium to an OD_540nm_ of 0.15–0.18; ~10^7^ CFU/ml) and incubated at 25 °C. Relative biofilm formation of strains was quantified using a crystal violet assay and measured at OD_600nm_ using either a DU800 spectrophotometer (Beckman Coulter, Inc., CA) or a fluorescent plate reader (BioTek Instruments Inc, Vermont), as previously described [42].

### Co-culture competition assays

Overnight monocultures of both lysogens were sub-cultured into fresh medium and grown to mid-log phase (ca. 1.0 × 10^8^ CFU/ml). Cells were harvested by gentle centrifugation (5000 × *g* for 10 min), rinsed with fresh media to remove unbound phages and resuspended in fresh media. Lysogens were mixed at a ratio of 1:1 at an OD_540_ of 0.17 (~10^7^ CFU/ml) and incubated at 25 °C, with shaking, for broth culture competition experiments. After 24 h, samples were collected for: (1) genomic DNA extraction from bacteria and viruses; (2) viable counts; and (3) plaque assays. The relative abundance of each strain was determined using qPCR of genomic DNA isolated from mixed cellular biomass collected by centrifugation, following procedures outlined above. A caveat to the estimates of host abundance: the qPCR primers target genes within the prophage. As such, they do not distinguish between integrated and nonintegrated phage. Virus particles were enumerated from cell-free filtrate (0.2 µm) as described above. Samples were first treated with Fermentas DNAse at 5 U/ml for 30 min at 37 °C to destroy any free genomic DNA from lysed host cells. DNAase was heat inactivated by incubation of samples at 65 °C for 10 min. For qPCR of phages, samples were heated for 10 min at 95 °C and 2.5 µl was used as template in 25 µl reactions. Co-culture biofilm experiments were performed in a similar fashion with the following exceptions: 100 µl of 1:1 lysogen mixtures were added to individual wells of a 96-well microtiter dish and incubated at 25 °C for 48 h. qPCR was performed using extracted DNA from microbial biomass. Due to the adherent nature of the biofilm matrix, it was not possible to separate cells from the matrix which contains free-phage particles.

### Environmental virome data

From the same induced *E. huxleyi* bloom from which CB-D and ɸ-A were isolated, a sample was collected immediately following collapse of the bloom for sequence characterization of viral particles. Details of the induced bloom are described in [43]; the sample analyzed for genetic analysis of the viral community was collected on day 15. Two liters of water were pre-filtered through a 1.2 µm low-protein-binding Durapore membrane filter (Millipore Corp) and subsequently concentrated to a final volume of ~50 ml using a Vivaflow 200 benchtop system, with a 50 kDa cut-off polyethersulfone membrane. Viruses were further concentrated by ultracentrifugation at 28,000 rpm, 10 °C for 2 h. The viral pellet was dissolved in 200 µL of SM buffer (0.1 M NaCl, 8 mM MgSO_4_·7H_2_O, 50 mM Tris-HCl, 0.005% (w/v) glycerin). Lysis of the viral particles was performed in freshly made lysis buffer (250 mM EDTA pH 8.0, 1% SDS, 1 mg/ml Proteinase K). The agarose plugs were run at pulse-ramps at 8–30 s for 24 h at 14 °C on a 1% w/v SeaKem GTG agarose (FMC, Rockland, Maine) gel in 1X TBE gel buffer using a Bio-Rad DR-II CHEF Cell (Bio-Rad, Richmond, CA, USA) electrophoresis unit. Gels were visualized and digitized using the Fujifilm imaging system, LAS-3000, and 8 bands of interest (ranging in size from ~35 to 485 kb) were excised. DNA was eluted from the PFGE agarose gel slices in 10,000 MWCO Spectra/Por, Regenerated Cellulose dialysis membranes (Spectrum Laboratories Inc.CA, USA) by electrophoresis in 1× TAE buffer (40 mM Tris-HCl, 1 mM EDTA, 40 mM acetic acid, pH 8.0) for 3 h at 70 V. Further concentration of the DNA was performed using Vivaspin 500 columns (Milipore Corp) according to the manufacturer’s protocol. Eluted DNA was amplified based on a linker-adapter PCR method using the WGA1 and Genome Plex WGA reamplification kit from Sigma (Sigma Aldrich, St Louis, MO, USA. Six separate WGA reactions were run and pooled before further processing. GenElute PCR Clean-Up Kit (Sigma Aldrich) was used for purification of the products that were then stored at −80 °C until sequencing at the Broad Institute under the Gordon and Betty Moore Foundation’s Marine Phage, Virus, and Virome Sequencing Project.

### Statistical analysis

RT-qPCR data analysis and the normalized relative transcript quantity were calculated using the qBASE method [44]. Student’s *t* tests were used to determine significant difference using GraphPad Prism (GraphPad Software, Inc.).

### Nucleotide sequence accession numbers

The genome sequence for *Sulfitobacter* sp. strain CB-A was deposited in GenBank under the accession number PYUG00000000. Genome sequences for ɸ-A and CB-D (with ɸ-D within) were previously reported as HQ332142 and NC_027299, respectively [35, 36]. Size-selected virome libraries were previously deposited into the NCBI Short Read Archive (SRA; PRJNA47483). The two libraries referenced in this paper, ~35 kb and ~75 kb size fractions, are designated as ME-08-09 and ME-08-08, respectively, in the SRA.

## Results

### Genome comparisons of temperate ɸ-A and prophage ɸ-D reveal high sequence similarity

**Fig. 1:**
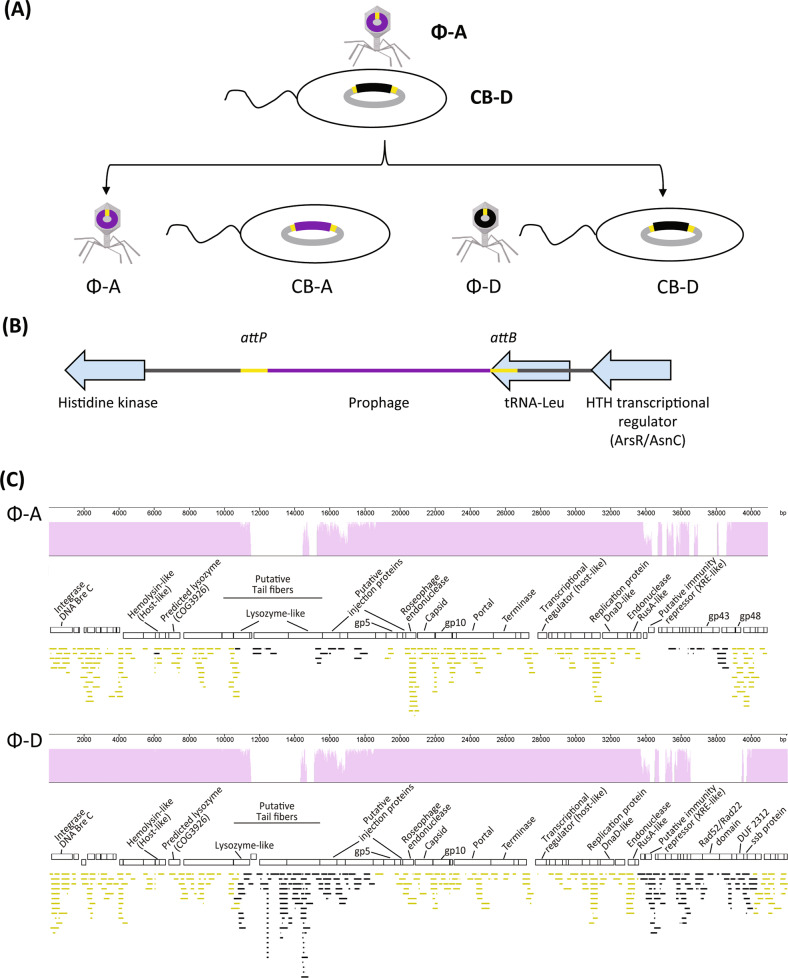
Overview of roseophage-host system. **a** Potential outcomes of superinfection of *Sulfitobacter* sp. strain CB-D with ɸ-A viral particles. **b** Within the CB-D genome, prophage D is flanked by 15 bp GC-rich direct terminal repeats (*attB*), within the 3′ end of a host tRNA-Leu gene. ɸ-A harbors a single copy of this sequence (termed *attP*). **c** Alignment of ɸ-A and ɸ-D genomes and recruitment of environmental virome reads. Purple plots show nucleotide identity between phage genomes (scale ranging from 0 to 100%). Open rectangles represent individual ORFs; predicted annotations are provided, where possible. Reads from a North Atlantic, size fractioned virome (~35 kb) (PRJNA47483) that mapped to either genome are shown directly beneath each phage ORF map. Reads were simultaneously recruited to both of the ɸ-A and ɸ-D genomes. Yellow bars represent reads that mapped to either ɸ-A and ɸ-D with equal fidelity (*n* = 562); the distribution of these reads across the two genomes was randomized. Those fragments in black are specific for either ɸ-A or ɸ-D (*n* = 161). Refer to Fig. S4 for the full suite of reads recruiting to each phage genome.

Within the genome of *Sulfitobacter* sp. CB-D lies the prophage ɸ-D, which is distinct from, yet highly similar to, ɸ-A, a temperate virus isolated from the same waters as CB-D (Fig. 1). Genome-wide nucleotide similarity alignment of ɸ-A and ɸ-D show that they share an average of 79% identity, with the majority of the genomes nearly identical. A CoreGenesUniqueGenes [45] analysis identified 58 highly homologous genes (BLASTp threshold score, 75) between ɸ-A and ɸ-D. Both phages carry a suite of genes for phage structure, replication/host regulations, host integration/excision, lysis/structure, and share the same DNA Bre-C-like integrase anticipated to facilitate integration into the host genome [35, 36].

The sequence variation between ɸ-A and ɸ-D is localized to two 4–6 kb regions, which primarily encode genes of unknown function, but also includes putative tail fibers protein genes, expected to be important for binding to host cell-surface receptors, and transcriptional regulators that may repress lytic genes during lysogeny. The putative transcriptional regulators encoded within each phage have low sequence identity to one another: <30% identity at amino acid level for all pairwise alignments. ɸ-D harbors two ORFs (SUFP_003; SUFP_050) that fall within the XRE transcriptional regulator superfamily. Both contain an XRE-family HTH domain but lack the *lexA*/signal peptidase superfamily domain common to characterized phage repressors within this family. ɸ-D also harbors an ORF (SUFP_063) with homology to the single stranded DNA binding protein family, with members involved in DNA replication via binding of ssDNA at the primosome assembly site. ɸ-A possesses two ORFs that belong to the XRE-superfamily and lack specific catalytic domains (SUFA_030 and SUFA_031).

### A temperate virus causes lytic infection and prophage induction

**Fig. 2:**
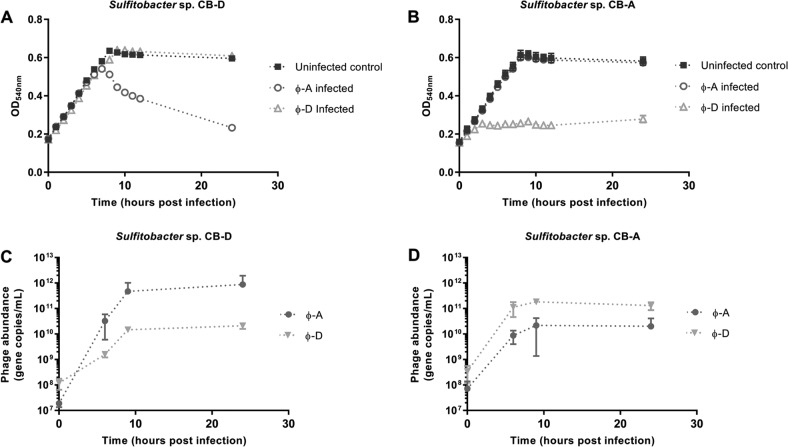
*Sulfitobacter* strains CB-D and CB-A susceptibility tests with ɸ-D and ɸ-A. **a** CB-D growth dynamics of cultures superinfected with ɸ-A (open circles), ɸ-D (open triangles), compared with uninfected controls (closed squares). **b** CB-A growth dynamics of cultures superinfected with ɸ-D (open triangles), ɸ-A (open circles), compared with uninfected controls (closed squares). Phage gene copies **c** during superinfection of CB-D with ɸ-A as shown in panel A and **d** during superinfection of CB-A with ɸ-D as shown in panel B, ɸ-A gene copies (closed circles) and ɸ-D gene copies (closed inverted triangles). Phage gene qPCR data represent sum of intracellular and extracellular gene copies. Averages of biological triplicates are reported for all treatments; technical triplicates were run for all qPCR assays. Error bars denote standard deviations and are obscured by the data markers in some instances. Data for this experiment are provided in Tables S4–S7.

Infection of *Sulfitobacter* sp. CB-D with the temperate phage ɸ-A results in prophage induction, production of both phage types and cell lysis. The growth dynamics of *Sulfitobacter* sp. CB-D cultures infected with ɸ-A are indistinguishable from uninfected controls until the onset of cell lysis at ~5 h post infection (h.p.i.), when significant differences in cultures are observed (Fig. 2a). By 10 h.p.i., optical densities in the infected cultures are ~40% of the uninfected controls and both phages are produced at unequal abundances (Fig. 2c). Infection of *Sulfitobacter* sp. CB-A with temperate phage ɸ-D also results in prophage induction, production of both phage types and cell lysis, with an apparent decrease in the latent period (3 h.p.i.) relative to CB-D ɸ-A superinfections (Fig. 2b, d). Using qPCR as a proxy for phage abundance, each of the superinfecting phages is present in at least tenfold higher abundance than the resident prophage 24 h.p.i. (Fig. 2c, d).

**Fig. 3:**
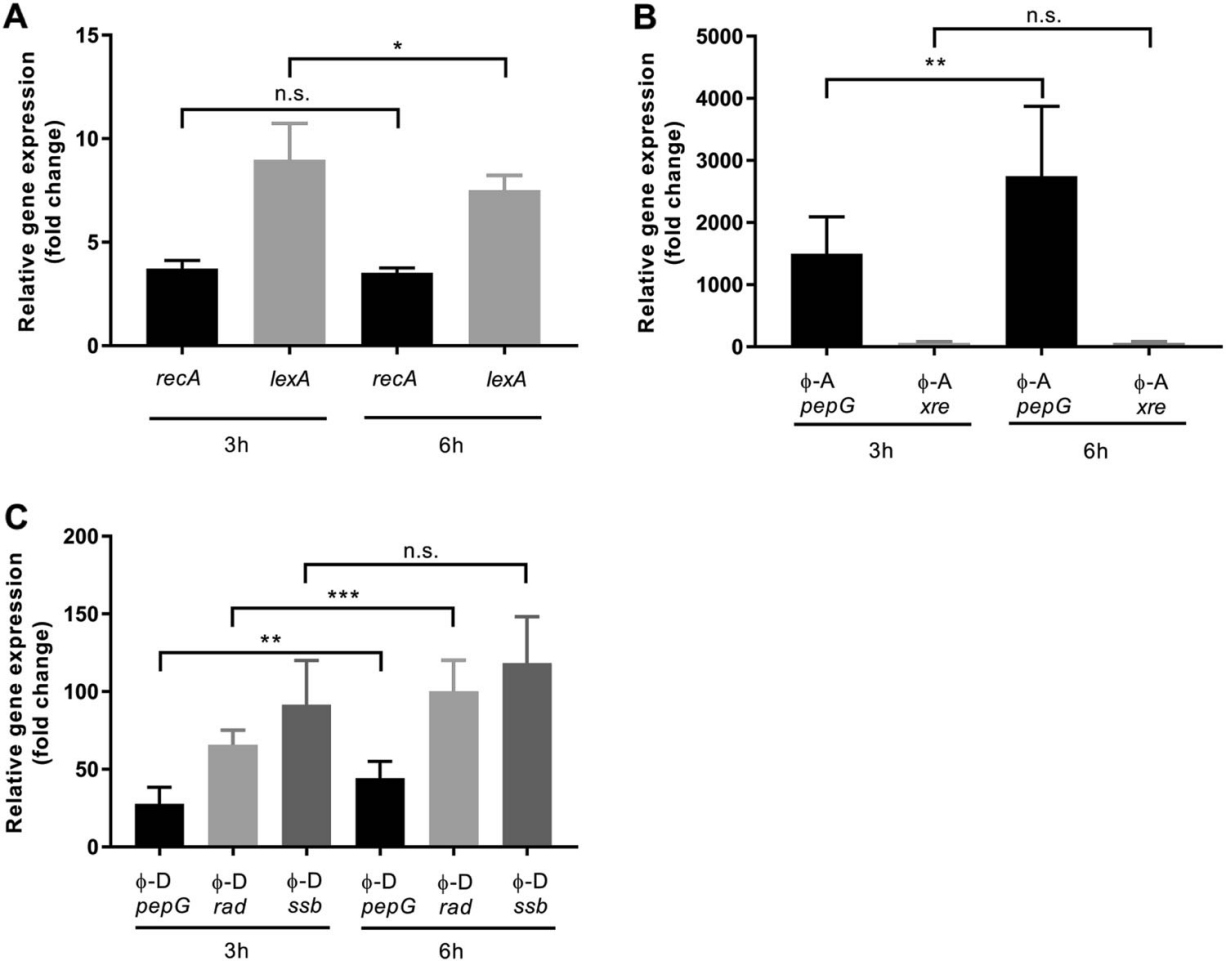
Relative gene expression of CB-D host and phage gene transcripts 3- and 6-h post superinfection with ɸ-A, relative to non-superinfected controls. **a** Fold change of host SOS response genes (*recA* and *lexA*). **b** Fold change of ɸ-A genes, peptidase (*pepG*) and XRE-like transcriptional regulator (*xre*). **c** Fold change of prophage (ɸ-D) genes, peptidase (*pepG*), ssDNA break repair protein (*rad52*), and DNA replication and repair protein (*ssb*). Significant differences (Student’s *t* tests) are denoted by asterisks (*p < 0.05; **p < 0.01; ***p < 0.001; n.s. not significant). Averages of biological and technical triplicates are reported for all treatments and error bars denote standard deviations. Data for this experiment are provided in Table S8.

Quantitative RT-PCR of genes diagnostic for the host *Sulfitobacter* sp. strain CB-D, ɸ-D, and ɸ-A provide further evidence that a mixed lytic-lysogenic infection occurs. Expression of host genes indicative of the SOS response, *recA* and *lexA*, as well as select phage genes involved in the presumptive lysogenic-lytic switch (XRE-type repressor [*rep/XRE*]), ɸ-D phage DNA replication and repair (single stranded DNA binding protein gene [*ssb*], ɸ-D double stranded DNA break repair gene [*rad52*]) and cell lysis (endolysin [*pepG*]) were monitored at two discrete time points preceding and following measurable culture lysis (3 and 6 h.p.i., respectively) (Fig. 3). Consistent with the differences in phage production, the relative increase of ɸ-A gene expression is greater than that observed for prophage (ɸ-D) genes (Fig. 3b, c). The ɸ-A XRE-type gene (SUAG_00031) is upregulated 66-fold 6 h.p.i. compared with an 8-fold expression increase of the ɸ-D *ssb* gene (SUFP050) at the same time point (Fig. 3b, c). Similarly, endolysin gene expression (SUAG_00073) of the superinfecting phage ɸ-A is upregulated 2700-fold 6 h.p.i, while ɸ-D endolysin gene expression (SUFP_019) is upregulated 44-fold at the same time point, relative to initial expression levels (Fig. 3b, c). Collectively, these data suggest both phages are actively employing lysogenic and lytic lifestyles that are likely influenced by the activities of the other.

### Generation of *Sulfitobacter* sp. strain CB-A

Within the genome of CB-D, the ɸ-D prophage is flanked by a 15 bp GC-rich direct terminal repeat (designated *attB*), within the 3′ end of a host tRNA-Leu gene (Fig. 1b), consistent with the observation that many phages and other genetic elements have high affinity for integration into tRNA genes [46]. ɸ-A harbors a single copy of this sequence, designated *attP*. Thus, we hypothesized that this putative attachment site could direct ɸ-A viral DNA to the appropriate integration site in its host, generating a new lysogen. To test this hypothesis, a superinfection experiment was conducted using ɸ-A and *Sulfitobacter* sp. strain CB-D. Four and 8 h post infection, aliquots of ɸ-A-infected *Sulfitobacter* sp. CB-D cultures were spread onto agar dishes. Fifty randomly selected colonies from each time point were then screened by PCR assay using phage-specific primers. Lysogens PCR positive for ɸ-A were recovered with relatively high frequency (~10% and ~20% of colonies at 4 h.p.i. and 8 h.p.i., respectively) (Table S2). In addition, this screening method provided evidence of transient polylysogens (PCR positive for both ɸ-A and ɸ-D). These putative polylysogens were not stably maintained, reverting to single-lysogens following three or fewer passages on fresh medium.

A representative ɸ-A positive strain, denoted CB-A, was selected for further study. Genome sequence analysis of CB-A revealed the integration of ɸ-A at the *attB* site. The ɸ-D prophage was not present, indicative of a substitution (Fig. 1c). Genome comparisons of CB-A and CB-D show that for all contigs to which CB-A Illumina reads mapped to the original CB-D genome, there are no nucleotide differences between these two strains outside of the regions of variation found in the prophages. The genome of CB-D was re-sequenced and showed no difference from the original sequence described in 2014 [36].

### Superinfection resistance and differing physiologies of lysogens

**Fig. 4:**
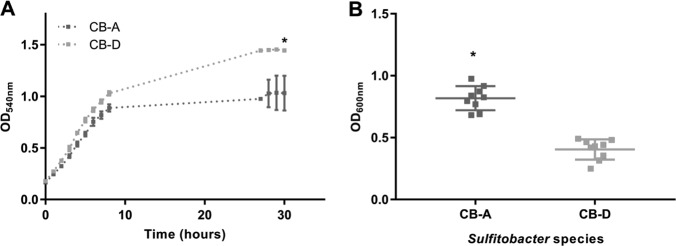
Physiological characteristics of CB-D and CB-A during different modes of growth. **a** Growth dynamics of CB-D (light gray) and CB-A (dark gray) in broth cultures and **b** biofilms at 24 h. Asterisks denote significant differences as determined by Student’s *t* tests (*p < 0.05). Averages of biological triplicates and technical triplicates are reported for all treatments. Error bars denote standard deviation and are obscured by the data markers in some instances. All data for this experiment are provided in Tables S9 and S10.

Each of the two lysogens demonstrates resistance to superinfection by the phage particles of the identical genotype: CB-D is resistant to infection with ɸ-D and CB-A is resistant to infection with ɸ-A. Yet each strain is susceptible to lytic infection by the other phage genotype which is accompanied by induction of the resident prophage (Figs. 2c, d and S1). In addition, under routine laboratory cultivation conditions the growth phenotypes of each lysogen were noticeably different. This qualitative assessment prompted quantitative phenotypic characterizations of these strains in liquid and surface-associated growth modes. In liquid culture, CB-D has a shorter generation time and greater maximum cell density than CB-A (Fig. 4a). Doubling times of the CB-D and CB-A lysogens were 75 and 100 min, respectively. In stationary phase, CB-A viable counts are half of CB-D (2.51 × 10^9^ [±7.00 × 10^8^] CFU/ml compared with 4.86 × 10^9^ [±1.48 × 10^8^] CFU/ml). In contrast, CB-A formed more robust biofilms relative to CB-D (Fig. 4b). This bulk measurement is supported by confocal microscopy images of CB-A and CB-D biofilms that show substantial differences in biofilm structure (Fig. S2). *Sulfitobacter* sp. strain CB-A has an average thickness (biomass) of 5.13 µm, compared with 3.48 µm for CB-D. The maximum biomass thickness was also larger for CB-A than CB-D and had a larger range (5.49–13.24 µm compared with 3.57–5.65 µm, respectively) (Fig. S3 and Table S3).

### Titers of free-phage suggest lysogens have different rates of spontaneous prophage induction

Given the discrepancy in growth dynamics of the two strains, we next determined whether there were quantifiable differences in free-phage titers in CB-A and CB-D cultures. CB-A stationary and mid-log phase cultures yielded 1.63 × 10^6^ (±2.08 × 10^5^) PFU/ml and 5.2 × 10^5^ (±8.5 × 10^4^) PFU/ml respectively. CB-D cultures of the same growth states do not yield quantifiable phage using plaque assays, indicating values below the 45 PFU/ml limit of detection for the assay.

### Prophage type determines outcome in head-to-head competition

**Fig. 5:**
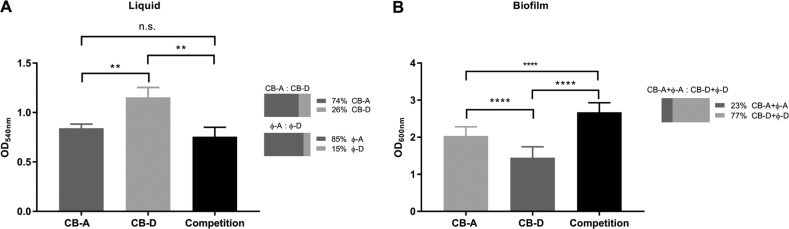
Head-to-head competition in liquid cultures and biofilms. **a** Final (24 h) culture densities of broth CB-A monocultures (dark gray), CB-D monocultures (light gray), and co-cultures (black). Horizontal bar graphs depict ratios of CB-D:CB-A and ɸ-A:ɸ-D in broth co-cultures as determined by qPCR. **b** Final (24 h) crystal violet biofilm assays for CB-A monocultures (dark gray), CB-D monocultures (light gray), and co-cultures (black) grown as biofilms. qPCR was used to quantify total number of gene copies of CB-D (+ɸ-D):CB-A (+ɸ-A) in co-culture, as represented in horizontal bar graph. Significant differences (Student’s *t* tests) are denoted by asterisks (*p < 0.05; **p < 0.01; ***p < 0.001; ****p < 0.0001; n.s. not significant). Averages of biological triplicates are reported for all treatments and error bars denote standard deviations. Technical triplicates were run for all qPCR assays and eight technical replicates were run for each biofilm assay. Data for this experiment are provided in Tables S11–S13.

The differences in spontaneous induction coupled with the susceptibility of each lysogen to infection by the opposing viral type, which in turn is accompanied by induction of the resident prophage, prompted us to perform competition experiments with these strains. In head-to-head competition (1:1 initial ratio) in broth culture, the ratio of CB-A to CB-D gene copies were 3.26 (range 2.00–4.73) after 24 h of co-culture. The co-cultures were ~90% and ~65% lower than typical densities for monocultures of CB-A and CB-D, respectively (Fig. 5a). The number of phage particles present in cell-free filtrates of these mixed cultures were six to one (ɸ-A:ɸ-D); ɸ-A and ɸ-D gene copies were 6.84 × 10^9^ (±2.18 × 10^9^) copies/ml and 1.18 × 10^9^ (±0.27 × 10^8^) copies/ml, respectively. Head-to-head competition assays during growth on a surface showed an opposite response: co-culture biofilms had 29% and 55% greater biomass than CB-A and CB-D monoculture biofilms, respectively (Fig. 5b). In addition, total ɸ-D gene copies were 3.4 times as high as ɸ-A (range 2.7–3.8; Fig. 5b); the biofilm matrix prevented physical separation of cells and unattached viruses, so summed values are presented.

### Environmental evidence for ɸ-A and ɸ-D

From the same induced *E. huxleyi* bloom from which CB-D and ɸ-A were originally isolated, samples were collected immediately following collapse of the phytoplankton bloom for genetic characterization of viral particles. Of the eight size-selected viral DNA fractions sequenced, reads from two libraries (~35 and ~75 kb size fractions) mapped to ɸ-A and ɸ-D genomes. From the ~35 kb library (*n* = 132,133 reads), 580 individual reads mapped to ɸ-A and 705 mapped to ɸ-D (Fig. S4). Of these, 562 reads mapped to homologous regions of both genomes. Unique reads mapped to the divergent regions of the ɸ-A (18 reads) and ɸ-D (143 reads) genomes (Fig. 1c). The small numbers of reads, 56 and 86, from the 75 kb library (*n* = 169,279 reads) that mapped to ɸ-A and ɸ-D, respectively, are likely the result of incomplete separation of DNA molecules during PFGE. These data indicate that viral particles from both phage types were present, at non-equal abundances, in natural populations.

## Discussion

Viral-mediated lysis of microbial cells in marine systems leads to quantitatively important impacts on food webs and biogeochemical cycles [47]. Yet, our understanding of marine host-virus interactions that give rise to host death, or otherwise influence host fitness, are limited. This is particularly true for temperate viruses of heterotrophic marine bacteria. Here, we describe a new marine host-phage system that advances our understanding of the complex interactions found amongst temperate phages and their susceptible hosts.

Lysogenized bacteria are typically resistant to superinfection, that is secondary infection, by homologous phages [reviewed in 48]. ɸ-A and ɸ-D are certainly homologous from a genomic perspective, showing a reasonably high degree of nucleotide identity across the full length of their ~40 kb genomes (79%) [36]. Yet, we demonstrate here these lysogens are susceptible to infection by a genetically similar phage. Consistent with the mosaicism commonly observed amongst related phage [49], the genomic differences between ɸ-A and ɸ-D are principally restricted to two 4–6 kb regions that appear to encode transcriptional regulators and tail fibers. Tail fiber adsorption to specific bacterial cell-surface receptors is the initial step in successful infection [50, 51]. Thus, differences in the primary sequence of the ɸ-A and ɸ-D tail fiber proteins may indicate distinct cell-surface targets for each of these phages. Furthermore, the putative transcriptional regulatory proteins encoded on ɸ-A and ɸ-D map to broad, but distinct, protein families indicating a likelihood for genotypic-phenotypic mismatching [i.e., development of immunity groups; e.g., 52–54] leading to the observed symmetrical infection profiles.

Infection of CB-D with ɸ-A leads to the simultaneous production of ɸ-A and ɸ-D, indicative of both lytic infection and prophage induction. While we do not yet know the proteins that mediate the lysogenic-lytic switch in this system, gene expression assays from infected cell populations support quantitative measurements of phage abundance. A putative peptidase (*pepG*) encoded by ɸ-A is upregulated during superinfection relative to non-superinfected CB-D controls. Similarly, upregulation of the CB-D host genes *recA* and *lexA* indicates activation of the global SOS response, which has been shown to mediate the lysogenic to lytic switch in various bacteria [e.g., *Salmonella enterica*, *Escherichia coli*, and *Pseudomonas aeruginosa*; reviewed in 55–57]. Indeed, superinfection by other phages has been shown to be a biotic factor influencing prophage induction, presumably through induction of the SOS response [58, 59]. In contrast, a ɸ-A putative transcriptional regulator (*xre*-like), is below the limit of detection, perhaps suggesting a role for this gene’s product in suppression of phage lytic genes during the lysogenic state. As the ɸ-A and ɸ-D encoded transcriptional regulators lack conserved catalytic domains common to well characterized phage repressors, these proteins may be valuable targets for future studies aimed at deciphering the lysogenic-lytic switch in these *Sulfitobacter-*phage pairs. An aspect of lytic activation of prophages in response to superinfection that has not been explored in our, or other systems, is whether lytic infection and prophage induction occur simultaneously in an individual cell or within distinct subpopulations of cells, one undergoing lytic infection by an exogenous phage and the other undergoing lytic activation of a previously quiescent prophage. It is possible that a subpopulation superinfected with one phage could communicate with non-superinfected counterparts, thus initiating a lytic induction, a phenomenon that has only recently been reported for a *Bacillus* phage [60].

In addition to a mixed infection resulting in the production of both ɸ-A and ɸ-D viral particles, infection of CB-D with ɸ-A also yields new lysogens in which the prophage appears to have been replaced by the superinfecting phage. The mechanism whereby this presumptive substitution occurs is not yet clear, but several possibilities exist. It could have been achieved through homologous recombination between the phage genomes, an oft-cited mechanism of viral evolution [61–63]. Alternatively, eviction of the prophage followed by integration of the ɸ-A genome could have led to the production of CB-A variants. While an intriguing possibility, there is presently little evidence in the literature to suggest such interactions occur amongst phages. A third possibility is that a subpopulation of host cells in our cultures lack a prophage, freeing the attachment site for integration. Using qPCR across the integration site, we estimate that a small subpopulation (ranging from 0.02 to 0.08% of *Sulfitobacter* sp. CB-D cultures) lack a prophage at the *attB* site (data not shown). Such individual cells would have increased susceptibility to lysogeny by ɸ-A invasion. Finally, integration of both prophages in tandem could result in the establishment of transient polylysogens. Due to an intrinsic instability arising from the high degree of nucleotide identity between the two phages, such presumed polylysogenic events might be expected to readily revert to a single phage type. Regardless of the apparent replacement mechanism, the relatively high frequency with which new lysogens are recovered from superinfections suggests either genotypic switching is prevalent with this two-phage-one-host system or that one-host-phage pair displays higher fitness than the other in a given environmental context.

Our data indicate a competitive interaction between the two host-phage pairs based on a fundamental difference in their lysogenic-lytic switches. One manifestation of these differences is altered frequencies of SPI that influence growth dynamics when the strains are cultivated planktonically and as biofilms. Rates of SPI are anticipated to be the combined result of stochasticity in gene expression (genetic noise) and induction of the SOS response. It has been observed that either a drop in phage repressor protein levels below a given threshold concentration or sporadic expression of integrase genes may initiate the lytic cycle [64]. Noise is pervasive in gene regulatory networks and can provide selective advantage to populations by increasing phenotypic heterogeneity within individual species and complex microbial communities [reviewed in 6]. Thus, prophages may exploit genetic noise to modulate the frequency of spontaneous activation. In contrast, the apparent instability of the lysogenic state in CB-A may indicate a nonoptimal pairing between host and phage. A recent example with nearly genetically identical marine Bacteriodetes strains reveals variation in infection efficiency across strains challenged with the same phage [65]. Regardless of the underlying mechanism(s) that give rise to the observed variation SPI in these two lysogens, this variation directly influences interactions amongst them.

Until recently, SPI was largely considered detrimental as some fraction of the cells is continuously lost by phage-induced lysis. However, benefits of SPI on bacterial fitness are now recognized, and include the release of extracellular DNA, which facilitates and enhances biofilm formation [6, 66], consistent with our findings. It has also been suggested that SPI dictates the maintenance and propagation of lysogeny in *S. enterica*, which is important for the evolution and diversity of host populations [67]. Relevant to our study is the notion that lysogens may use SPI as a form of specialized weaponry against susceptible cell types, whether they be nonlysogenized variants of the same strain or different species [14]. As an ecological adaptation to physically structured environments, temperate phages are theorized to enhance the fitness of their hosts through killing of susceptible competitors [68, 69]. Empirical studies support this proposed form of allelopathy. For example, *S. enterica* studies demonstrate selective eradication of nonimmune hosts in mixed populations as a means of a competing strategy by Gifsy 2 lysogenized strains [67]. Rat model competition studies show that *P. aeruginosa* strains lysogenized by Liverpool Epidemic Strain prophages use the phages as anti-competitor weapons against phage-susceptible *P. aeruginosa* populations in a chronic lung infection model [70]. Studies using *E. coli* MG1655 lysogenized with *λ* reveal the competitive nature of this type of inaction is anticipated to be limited, as lysogenization of susceptible hosts ultimately diminishes nonlysogenized “competitors” [71]. Finally, in a more complicated scenario involving members of the microbiome of the freshwater metazoan, *Hydra vulgaris*, one microbiome member, a *Curvibacter* species, possesses an inducible prophage that lytically infects another microbiome member, a *Duganella* strain. Mathematical modeling predicts this interaction may modulate competition amongst microbiome members [72].

Our system proposes a new element to this type of interaction: the reciprocal attack by genetically similar phages that share an integration site in a common host. Head-to-head competition experiments between CB-A and CB-D indicate different fates depending upon mode of bacterial growth: planktonic or biofilm, two modes in which Roseobacters, in general, and *Sulfitobacters*, in particular, thrive in nature [73, 74]. We acknowledge these laboratory-based experiments are unlikely to be a faithful reflection of the interactions these host-phage pairs would display in the wild, where, amongst other factors, nutrients and cell abundances would be lower and community complexity higher, respectively. However, genetic signatures identified in a virome collected at the terminus of the phytoplankton bloom in the North Atlantic from which the original host and phage were isolated supports the co-existence of both phage particles in natural waters, at nonequivalent abundances (i.e., ɸ-D particles are better represented than ɸ-A). At the time of sample collection, phytoplankton debris was elevated in this system, as were Roseobacter abundances [75]. The overrepresentation of ɸ-D relative to ɸ-A appears consistent with our laboratory co-culture biofilm experiments in which the ɸ-D type prevails.

The lab and field data presented here demonstrate that both phage types can occur in mixed populations and indicate the competitiveness of a given host-virus pair is niche specific. Thus, the maintenance of both phage types within a population may be advantageous to a given host over multiple generations and across marine landscapes. Indeed, we might consider these discrete host-phage populations as analogous to bacterial populations that exhibit phase variation. Phase variation has been described as an interchange between physiological “states”, and is exemplified by the production of antigenic components, H1 and H2, in motile and nonmotile strains of *S. enterica* (formerly known as *Salmonella choleraesuis*) that allow the bacterium to rapidly adapt to shifting environmental conditions [76]. Why co-cultures of CB-A and CB-D have divergent outcomes depending upon growth mode (planktonic vs. biofilm) is presently unknown. However, given that integrated viral genes within a lysogen can confer a myriad of phenotypic and fitness effects (e.g., increased antibiotic resistance, biofilm formation ability, altered growth dynamics, metabolic reprograming [2, 68, 77, 78]), it is intriguing to consider that the physiological distinctions displayed by CB-A and CB-D extend beyond variation in their lysogenic-lytic switch mechanisms. Important next steps in this research also include development of tools to allow the tracking of individual cells and viral particles to better elucidate the dynamics of each of the players in this complex interaction.

Lysogeny is widespread in nature and has recently received considerable attention in the context of marine systems where focus has been on elucidation of the environmental factors that drive temperate phage into either a lytic or lysogenic state [e.g., 27, 28]. Our work reveals the importance of intrinsic factors in influencing host-phage interactions and highlights the value of considering states that lie between the bilateral viewpoint of wholesale lysogeny or rampant lysis within a population. Characterization of a two-phage-one-host model system suggests new mechanisms of microbial competition and cooperation in which host-phage pairs may form coalitions to challenge one another. The outcomes of these “challenges” appear context dependent, and may to lead to niche-specific quasi-state equilibria. The extent to which these cooperative behaviors are influenced by other environmental factors (e.g., nutrients, temperature, and host abundance), include additional lysogen-state phenotypic differences and/or modulate community composition remains to be determined. Another open question is the prevalence of these types of host-phage interactions in marine systems. Modern abilities to sequence the genomic content of individual cells and free viruses in a culture-independent manner (e.g., [79, 80]) should facilitate studies aimed at determining whether interactions such as those described here occur in nature. Given the extent of genetic microheterogeneity present in both marine microbial and viral communities, we predict these types of coalitions represent an overlooked component of host-phage interactions in the seas, particularly in environments where the chemical and physical properties undergo dramatic and rapid change (e.g., phytoplankton blooms).

## Supplementary information

Supplemental materials
