# Supplementary material for: Genetically similar temperate phages form coalitions with their shared host that lead to niche-specific fitness effects
Source: ISME J. 2020 Apr 2;14(7):1688–700. doi: 10.1038/s41396-020-0637-z (PMC7305329; doi:10.1038/s41396-020-0637-z)
Supplement: Supplementary file 1 — Supplemental materials [file 41396_2020_637_MOESM1_ESM.docx]

**Supplementary information**

**Genetically similar temperate phages differentially affect the physiology of their shared host**

Jonelle T. Basso^1^, Nana Y. D. Ankrah^1ǂ^, Matthew J. Tuttle^1^, Alex S. Grossman^1^, Ruth-Anne Sandaa^2^ and Alison Buchan^1*^

^1^University of Tennessee Knoxville, Department of Microbiology, 1311 Cumberland Avenue, 307 Ken and Blaire Mossman Bldg., Knoxville TN 37996, USA.

^2^University of Bergen, Department of Biological Sciences, PO 7803, N-5020, Bergen, Norway

ǂ Current address: 5136 Comstock Hall, Cornell University, Ithaca, NY, 14853

* Corresponding author: University of Tennessee Knoxville, Department of Microbiology, 1311 Cumberland Avenue, 307 Ken and Blaire Mossman Bldg., Knoxville TN 37996, USA. Tel: 865-974-5234; Email: [abuchan@utk.edu](mailto:abuchan@utk.edu)

| **Table S1. Oligonucleotides used in this study** | | | |
| --- | --- | --- | --- |
| **Primer name** | **Primer sequence (5'-3')** | **Gene target** | **Purpose/experiment** |
| alaS_577for | GGAGAGAGTGACGCATGGAT | alanine tRNA hydrolase | Host reference gene |
| alaS_719rev | CAGCTGTCGAGATGGACGTA | alanine tRNA hydrolase | Host reference gene |
| map_164for | TCACGCAGATGATCGAAGAC | methionine aminopeptidase | Host reference gene |
| map_334rev | CATCGACAATTACGGTGACG | methionine aminopeptidase | Host reference gene |
| rpoC_3196for | AAAAAGTCCGTCGTGGTGAC | RNA polymerase | Host reference gene |
| rpoC_3356rev | AACGGCATGTCTTCCATAGG | RNA polymerase | Host reference gene |
| 2047LexA_for | ACTCGATCTGCTGGCCTTTA | *lexA* transcriptional regulator | Host SOS response |
| 2047LexA_rev | TCGGGCAGTTTCACTACCTC | *lexA* transcriptional regulator | Host SOS response |
| 2047RecA_for | CTGATTTCCCAGCCTGACAC | recombinase *recA* | Host SOS response |
| 2047RecA_rev | AGCCTGTCAGCTTACGCATT | recombinase *recA* | Host SOS response |
| Pro PEPG_for | CTATGAAGGCATGGGCGATA | endolysin | ɸ-NYA Induction |
| Pro PEPG_rev | GGCGATCGATCCAACACT | endolysin | ɸ-NYA Induction |
| Pro Rad_for | TGGCCCTCTACGACAAAGAC | Rad52/22 dsDNA break repair | ɸ-NYA Induction |
| Pro Rad_rev | TCGTTTAGTTCGTGCTGCAT | Rad52/22 dsDNA break repair | ɸ-NYA Induction |
| Pro SSB_for | GCCTTGGAACGTCAATTCAT | ssDNA binding protein (*ssb*) | ɸ-NYA Induction |
| Pro SSB_rev | ACAACGGCAAGGACAAGAAC | ssDNA binding protein (*ssb*) | ɸ-NYA Induction |
| Pro rep_rev | CCGTGCCATTATTTGGCTAT | phage repressor | ɸ-NYA Induction |
| Pro rep_rev | GCTAATGTGCTGGGCCTTAG | phage repressor | ɸ-NYA Induction |
| int772_for | TGGGTCATTCTAACGCTGGT | integrase | Phage lysogeny establishment |
| int937_rev | ATTCCACAATCTCAAGCGCC | integrase | Phage lysogeny establishment |
| 47AHTH_XRE_for | CAAAAGCTGACGCAGACTCA | phage repressor | ɸ-47A lysogeny establishment |
| 47AHTH_XRE_rev | ATATCCCGCATCAGCTCAAC | phage repressor | ɸ-47A lysogeny establishment |
| 47A PEPG_for | GTGTTTGCATAATCGGCAAG | endolysin | ɸ-47A replication |
| 47A PEPG_rev | CGGATCTGGAAAACCAGCTT | endolysin | ɸ-47A replication |
| 2047PP1_for | TATTCATAGCGAGGCGCAGT | endolysin | ɸ-NYA enumeration |
| 2047PP1_rev | ATACCTGCCCAACGTCACAG | endolysin | ɸ-NYA enumeration |
| 2047A-C_for | CCCATGTGTATGTCGCCTCT | endolysin | ɸ-47A enumeration |
| 2047A-C_rev | CAGCGTTGAAAAAGGCTCTG | endolysin | ɸ-47A enumeration |
| jxn761U_for | GGCCAGCATAACCGTTTCC | histidine kinase | Phage Integration site identification |
| int937_rev | ATTCCACAATCTCAAGCGCC | integrase | Phage Integration site identification |
| jxn1105D_rev | TGTCGCCAACACCTCTACC | HTH transcriptional regulator | Phage Integration site identification |
| jxn1105D_for | GGGAGGCATGAGCGTAGAA | hypothetical protein | Phage Integration site identification |

| **Table S2. Frequency (percentage) of ɸ-A PCR positive colonies from superinfection studies (MOI 0.06)** | | | | |
| --- | --- | --- | --- | --- |
| **Time (h.p.i.)** | **Replicate 1** | **Replicate 2** | **Replicate 3** | **Average** |
| 4 | 0 | 5 | 20 | 8 |
| 8 | 15 | 10 | 30 | 18 |

**Table S3. Comstat2 data analysis**

|  | **Biomass** | **Maximum thickness** | **Roughness coefficient** | **Thickness distribution** |  |
| --- | --- | --- | --- | --- | --- |
| **Organism name and Z stack field of view number** | **Biomass (µm^3/µm^2):** | **Maximum thickness (µm):** | **Roughness Coefficient (Ra*):** | **Average thickness (Entire area) (µm):** | **Average thickness (Biomass) (µm):** |
| *Sulfitobacter* sp. strain CB-A. Z stack field of view: 1 | 1.18876 | 5.4879 | 1.00278 | 2.46208 | 4.91842 |
| *Sulfitobacter* sp. strain CB-A. Z stack field of view: 2 | 1.46763 | 6.1336 | 1.00804 | 2.03385 | 3.78813 |
| *Sulfitobacter* sp. strain CB-A. Z stack field of view: 3 | 1.71986 | 5.4879 | 0.7344 | 3.01986 | 4.72083 |
| *Sulfitobacter* sp. strain CB-A. Z stack field of view: 4 | 0.11164 | 13.2356 | 1.90794 | 0.32584 | 7.07645 |
|  |  |  |  |  |  |
| *Sulfitobacter* sp. strain CB-D. Z stack field of view: 1 | 0.53438 | 3.5706 | 1.28152 | 0.63001 | 1.73872 |
| *Sulfitobacter* sp. strain CB-D. Z stack field of view: 2 | 0.32996 | 5.6534 | 1.73059 | 0.60152 | 4.44958 |
| *Sulfitobacter* sp. strain CB-D. Z stack field of view: 3 | 0.53916 | 5.6534 | 1.36748 | 0.74707 | 2.34794 |
| *Sulfitobacter* sp. strain CB-D. Z stack field of view: 4 | 0.22374 | 5.6534 | 1.73358 | 0.66207 | 4.95401 |
| *Sulfitobacter* sp. strain CB-D. Z stack field of view: 5 | 2.31132 | 5.6534 | 0.22504 | 4.5969 | 4.82112 |
| *Sulfitobacter* sp. strain CB-D. Z stack field of view: 6 | 0.34321 | 5.6534 | 1.56043 | 0.39356 | 1.78002 |
| *Sulfitobacter* sp. strain CB-D. Z stack field of view: 7 | 0.14941 | 5.6534 | 1.85548 | 0.29573 | 4.09248 |
| *Sulfitobacter* sp. strain CB-D. Z stack field of view: 8 | 0.32476 | 5.6534 | 1.68187 | 0.73548 | 4.58514 |
| *Sulfitobacter* sp. strain CB-D. Z stack field of view: 9 | 0.31593 | 5.6534 | 1.62062 | 0.47826 | 2.51737 |

**Table S4. Optical density (OD 540 nm) data for ɸ-A infected, ɸ-D infected, and uninfected, *Sulfitobacter* sp. CB-D cells as shown in Fig 2A**

|  | Control | | | | |  | Φ-A Infected | | | | |  | Φ-D Infected | | | | |
| --- | --- | --- | --- | --- | --- | --- | --- | --- | --- | --- | --- | --- | --- | --- | --- | --- | --- |
| Time p.i. (h) | Rep 1 | Rep 2 | Rep 3 | Average | Std dev |  | Rep 1 | Rep 2 | Rep 3 | Average | Std  Dev |  | Rep 1 | Rep 2 | Rep 3 | Average | Std dev |
| 0 | 0.170 | 0.170 | 0.173 | 0.171 | 0.002 |  | 0.183 | 0.172 | 0.171 | 0.175 | 0.007 |  | 0.171 | 0.170 | 0.172 | 0.171 | 0.001 |
| 1 | 0.238 | 0.234 | 0.237 | 0.236 | 0.002 |  | 0.247 | 0.237 | 0.234 | 0.239 | 0.007 |  | 0.221 | 0.220 | 0.219 | 0.220 | 0.001 |
| 2 | 0.291 | 0.294 | 0.289 | 0.291 | 0.003 |  | 0.300 | 0.286 | 0.285 | 0.290 | 0.008 |  | 0.274 | 0.273 | 0.272 | 0.273 | 0.001 |
| 3 | 0.348 | 0.346 | 0.350 | 0.348 | 0.002 |  | 0.362 | 0.340 | 0.341 | 0.348 | 0.012 |  | 0.327 | 0.323 | 0.325 | 0.325 | 0.002 |
| 4 | 0.411 | 0.411 | 0.414 | 0.412 | 0.002 |  | 0.426 | 0.404 | 0.411 | 0.414 | 0.011 |  | 0.390 | 0.384 | 0.386 | 0.387 | 0.003 |
| 5 | 0.482 | 0.480 | 0.479 | 0.480 | 0.002 |  | 0.484 | 0.462 | 0.466 | 0.471 | 0.012 |  | 0.456 | 0.451 | 0.452 | 0.453 | 0.003 |
| 6 | 0.537 | 0.541 | 0.536 | 0.538 | 0.003 |  | 0.524 | 0.506 | 0.505 | 0.512 | 0.011 |  | 0.509 | 0.505 | 0.506 | 0.507 | 0.002 |
| 7 | 0.585 | 0.581 | 0.577 | 0.581 | 0.004 |  | 0.554 | 0.532 | 0.538 | 0.541 | 0.011 |  | 0.551 | 0.546 | 0.545 | 0.547 | 0.003 |
| 8 | 0.636 | 0.631 | 0.640 | 0.636 | 0.005 |  | 0.527 | 0.503 | 0.506 | 0.512 | 0.013 |  | 0.598 | 0.586 | 0.584 | 0.589 | 0.008 |
| 9 | 0.630 | 0.626 | 0.625 | 0.627 | 0.003 |  | 0.457 | 0.438 | 0.439 | 0.445 | 0.011 |  | 0.643 | 0.645 | 0.630 | 0.639 | 0.008 |
| 10 | 0.622 | 0.614 | 0.618 | 0.618 | 0.004 |  | 0.431 | 0.410 | 0.413 | 0.418 | 0.011 |  | 0.640 | 0.634 | 0.642 | 0.639 | 0.004 |
| 11 | 0.613 | 0.617 | 0.618 | 0.616 | 0.003 |  | 0.410 | 0.397 | 0.392 | 0.400 | 0.009 |  | 0.637 | 0.624 | 0.634 | 0.632 | 0.007 |
| 12 | 0.612 | 0.613 | 0.616 | 0.614 | 0.002 |  | 0.398 | 0.380 | 0.377 | 0.385 | 0.011 |  | 0.637 | 0.624 | 0.633 | 0.631 | 0.007 |
| 24 | 0.595 | 0.594 | 0.599 | 0.596 | 0.003 |  | 0.248 | 0.231 | 0.221 | 0.233 | 0.014 |  | 0.614 | 0.604 | 0.609 | 0.609 | 0.005 |

**Table S5. Optical density (OD 540 nm) data for ɸ-A infected, ɸ-D infected, and uninfected, *Sulfitobacter* sp. CB-A cells as shown in Fig 2B**

|  | Control | | | | |  | Φ-A Infected | | | | |  | Φ-D Infected | | | | |
| --- | --- | --- | --- | --- | --- | --- | --- | --- | --- | --- | --- | --- | --- | --- | --- | --- | --- |
| Time p.i. (h) | Rep 1 | Rep 2 | Rep 3 | Uninfected controls | Stdv |  | Rep 1 | Rep 2 | Rep 3 | Φ-A infected | Stdv |  | Rep 1 | Rep 2 | Rep 3 | Φ-D infected | Stdv |
| 0 | 0.151 | 0.153 | 0.175 | 0.16 | 0.013 |  | 0.154 | 0.153 | 0.156 | 0.154 | 0.002 |  | 0.154 | 0.155 | 0.157 | 0.155 | 0.002 |
| 1 | 0.213 | 0.214 | 0.244 | 0.224 | 0.018 |  | 0.214 | 0.215 | 0.216 | 0.215 | 0.001 |  | 0.192 | 0.186 | 0.189 | 0.189 | 0.003 |
| 2 | 0.263 | 0.262 | 0.292 | 0.272 | 0.017 |  | 0.263 | 0.269 | 0.268 | 0.267 | 0.003 |  | 0.226 | 0.224 | 0.223 | 0.224 | 0.002 |
| 3 | 0.323 | 0.319 | 0.349 | 0.33 | 0.016 |  | 0.319 | 0.323 | 0.326 | 0.323 | 0.004 |  | 0.254 | 0.254 | 0.256 | 0.255 | 0.001 |
| 4 | 0.385 | 0.381 | 0.415 | 0.394 | 0.019 |  | 0.38 | 0.382 | 0.386 | 0.383 | 0.003 |  | 0.247 | 0.243 | 0.249 | 0.246 | 0.003 |
| 5 | 0.452 | 0.45 | 0.48 | 0.461 | 0.017 |  | 0.447 | 0.439 | 0.452 | 0.446 | 0.007 |  | 0.245 | 0.245 | 0.241 | 0.244 | 0.002 |
| 6 | 0.502 | 0.497 | 0.536 | 0.512 | 0.021 |  | 0.494 | 0.499 | 0.502 | 0.498 | 0.004 |  | 0.251 | 0.256 | 0.248 | 0.252 | 0.004 |
| 7 | 0.547 | 0.535 | 0.577 | 0.553 | 0.022 |  | 0.54 | 0.54 | 0.543 | 0.541 | 0.002 |  | 0.247 | 0.267 | 0.242 | 0.252 | 0.013 |
| 8 | 0.604 | 0.594 | 0.642 | 0.613 | 0.025 |  | 0.601 | 0.595 | 0.602 | 0.599 | 0.004 |  | 0.243 | 0.271 | 0.255 | 0.256 | 0.014 |
| 9 | 0.609 | 0.597 | 0.637 | 0.614 | 0.021 |  | 0.598 | 0.596 | 0.612 | 0.602 | 0.009 |  | 0.261 | 0.279 | 0.258 | 0.266 | 0.011 |
| 10 | 0.592 | 0.589 | 0.63 | 0.604 | 0.023 |  | 0.586 | 0.594 | 0.597 | 0.592 | 0.006 |  | 0.235 | 0.264 | 0.247 | 0.249 | 0.015 |
| 11 | 0.59 | 0.586 | 0.62 | 0.599 | 0.019 |  | 0.581 | 0.591 | 0.596 | 0.589 | 0.008 |  | 0.255 | 0.246 | 0.234 | 0.245 | 0.011 |
| 12 | 0.586 | 0.582 | 0.625 | 0.598 | 0.024 |  | 0.582 | 0.585 | 0.595 | 0.587 | 0.007 |  | 0.242 | 0.243 | 0.253 | 0.246 | 0.006 |
| 24 | 0.579 | 0.562 | 0.605 | 0.582 | 0.022 |  | 0.562 | 0.591 | 0.566 | 0.573 | 0.016 |  | 0.258 | 0.292 | 0.285 | 0.278 | 0.018 |

**Table S6. qPCR data (A phage primer set) for data shown in Fig 2C and D.**

| Primer AC._ | | | | | | | | | | |
| --- | --- | --- | --- | --- | --- | --- | --- | --- | --- | --- |
|  |  |  | Rep A | |  | Rep B | |  | Rep C | |
|  |  | Time | average | Stdv |  | average | Stdv |  | average | Stdv |
| CB-D | A only | T0 | 2.54E+07 | 3.35E+06 |  | 1.76E+07 | 3.83E+06 |  | 1.36E+07 | 4.14E+06 |
|  |  | T6 | 6.27E+10 | 3.71E+09 |  | 1.26E+10 | 3.10E+09 |  | 2.21E+10 | 1.73E+09 |
|  |  | T9 | 1.08E+12 | 9.20E+10 |  | 1.17E+11 | 2.14E+10 |  | 1.93E+11 | 7.07E+09 |
|  |  | T24 | 2.13E+12 | 2.88E+11 |  | 1.86E+11 | 1.31E+10 |  | 3.52E+11 | 5.09E+10 |
| CB-A | A only | T0 | 1.92E+08 | 2.05E+07 |  | 1.32E+08 | 1.58E+07 |  | 7.09E+07 | 5.95E+06 |
|  |  | T6 | 2.96E+08 | 4.53E+07 |  | 2.57E+08 | 5.41E+07 |  | 1.81E+08 | 2.49E+07 |
|  |  | T9 | 1.63E+08 | 3.44E+07 |  | 7.47E+07 | 2.18E+07 |  | 8.14E+07 | 2.15E+07 |
|  |  | T24 | 1.61E+08 | 1.01E+07 |  | 9.69E+07 | 2.99E+07 |  | 4.40E+07 | 5.36E+06 |
| CB-A | Control | T0 | 1.66E+08 | 1.37E+07 |  | 9.28E+07 | 2.65E+07 |  | 4.99E+07 | 1.82E+06 |
|  |  | T6 | 3.68E+08 | 1.91E+07 |  | 1.84E+08 | 1.88E+07 |  | 2.53E+08 | 1.52E+07 |
|  |  | T9 | 1.06E+08 | 5.31E+06 |  | 9.38E+07 | 8.96E+06 |  | 5.15E+07 | 6.81E+06 |
|  |  | T24 | 9.62E+07 | 1.34E+07 |  | 5.69E+07 | 3.38E+06 |  | 2.97E+07 | 3.47E+06 |
| CB-A | D only | T0 | 1.49E+08 | 3.80E+07 |  | 3.21E+07 | 5.07E+06 |  | 3.40E+07 | 4.79E+06 |
|  |  | T6 | 1.36E+10 | 5.45E+08 |  | 7.78E+09 | 1.22E+09 |  | 4.61E+09 | 7.71E+07 |
|  |  | T9 | 4.54E+10 | 3.83E+09 |  | 7.23E+09 | 1.67E+09 |  | 1.30E+10 | 2.12E+09 |
|  |  | T24 | 4.43E+10 | 2.46E+09 |  | 8.87E+09 | 2.02E+09 |  | 6.99E+09 | 1.51E+08 |

**Table S7. qPCR data (D phage primer set) for data shown in Fig 2C and D.**

| Primer PP1 | | | | | | | | | | |
| --- | --- | --- | --- | --- | --- | --- | --- | --- | --- | --- |
|  |  |  | Rep A | |  | Rep B | |  | Rep C | |
|  |  | time | average | Stdv |  | average | Stdv |  | average | Stdv |
| CB-D | A only | T0 | 1.74E+08 | 1.54E+07 |  | 5.97E+07 | 7.96E+06 |  | 1.31E+08 | 3.69E+07 |
|  |  | T6 | 1.97E+09 | 5.14E+08 |  | 1.21E+09 | 7.53E+07 |  | 1.65E+09 | 1.23E+08 |
|  |  | T9 | 1.49E+10 | 2.71E+09 |  | 1.68E+10 | 6.32E+08 |  | 1.18E+10 | 3.23E+08 |
|  |  | T24 | 2.37E+10 | 2.76E+09 |  | 2.38E+10 | 3.59E+09 |  | 1.53E+10 | 8.55E+08 |
| CB-D | Control | T0 | 2.40E+08 | 2.66E+07 |  | 6.59E+07 | 6.65E+06 |  | 1.18E+08 | 7.95E+06 |
|  |  | T6 | 4.91E+08 | 1.08E+08 |  | 1.10E+08 | 1.53E+07 |  | 2.91E+08 | 4.22E+07 |
|  |  | T9 | 1.53E+08 | 3.11E+06 |  | 9.39E+07 | 1.26E+07 |  | 7.25E+07 | 7.97E+06 |
|  |  | T24 | 1.41E+08 | 1.42E+07 |  | 9.70E+07 | 6.63E+06 |  | 9.14E+07 | 6.05E+06 |
| CB-D | D only | T0 | 1.22E+09 | 8.24E+07 |  | 3.26E+08 | 5.17E+07 |  | 4.65E+08 | 5.27E+07 |
|  |  | T6 | 1.52E+09 | 1.31E+08 |  | 4.25E+08 | 7.62E+07 |  | 5.85E+08 | 1.06E+08 |
|  |  | T9 | 1.05E+09 | 2.23E+08 |  | 5.70E+08 | 5.71E+07 |  | 4.86E+08 | 5.23E+07 |
|  |  | T24 | 1.17E+09 | 7.96E+07 |  | 6.77E+08 | 1.03E+08 |  | 4.48E+08 | 9.67E+07 |
| CB-A | D only | T0 | 5.78E+08 | 6.59E+07 |  | 1.53E+08 | 1.28E+07 |  | 2.85E+08 | 3.16E+07 |
|  |  | T6 | 1.88E+11 | 2.75E+10 |  | 7.36E+10 | 4.61E+09 |  | 7.52E+10 | 1.23E+09 |
|  |  | T9 | 1.78E+11 | 4.15E+10 |  | 1.77E+11 | 1.63E+10 |  | 1.94E+11 | 2.46E+10 |
|  |  | T24 | 1.43E+11 | 3.55E+10 |  | 1.65E+11 | 1.43E+10 |  | 8.34E+10 | 6.09E+08 |

**Table S8. RT qPCR data for Fig 3**

| **phiA infected CB-D cultures** | | | | |  | |  |  |  |  |  |
| --- | --- | --- | --- | --- | --- | --- | --- | --- | --- | --- | --- |
| time point  (-rep) | | gene | C(t) | time point  (-rep) | gene | | C(t) | time point  (-rep) | gene | C(t) |  |
| T0-1 | | 1 map | 23.03 | T3-1 | 1 map | | 22.9 | T6-1 | 1 map | 27.83 |  |
| T0-1 | | 1 map | 22.49 | T3-1 | 1 map | | 22.5 | T6-1 | 1 map | 27.68 |  |
| T0-1 | | 1 map | 22.33 | T3-1 | 1 map | | 22.5 | T6-1 | 1 map | 27.5 |  |
| T0-1 | | 2 alaS | 23.67 | T3-1 | 2 alaS | | 23.3 | T6-1 | 2 alaS | 28.03 |  |
| T0-1 | | 2 alaS | 23.49 | T3-1 | 2 alaS | | 23.2 | T6-1 | 2 alaS | 27.8 |  |
| T0-1 | | 2 alaS | 23.10 | T3-1 | 2 alaS | | 22.9 | T6-1 | 2 alaS | 27.77 |  |
| T0-1 | | 3 rpoC | 17.99 | T3-1 | 3 rpoC | | 20.1 | T6-1 | 3 rpoC | 24.32 |  |
| T0-1 | | 3 rpoC | 17.68 | T3-1 | 3 rpoC | | 20 | T6-1 | 3 rpoC | 24.22 |  |
| T0-1 | | 3 rpoC | 17.66 | T3-1 | 3 rpoC | | 20.1 | T6-1 | 3 rpoC | 24.27 |  |
| T0-1 | | 4 RecA | 21.80 | T3-1 | 4 RecA | | 20.5 | T6-1 | 4 RecA | 25.36 |  |
| T0-1 | | 4 RecA | 21.69 | T3-1 | 4 RecA | | 20.1 | T6-1 | 4 RecA | 25.13 |  |
| T0-1 | | 4 RecA | 21.54 | T3-1 | 4 RecA | | 20 | T6-1 | 4 RecA | 25.09 |  |
| T0-1 | | 5 LexA | 24.65 | T3-1 | 5 LexA | | 22.1 | T6-1 | 5 LexA | 26.74 |  |
| T0-1 | | 5 LexA | 24.05 | T3-1 | 5 LexA | | 21.4 | T6-1 | 5 LexA | 26.45 |  |
| T0-1 | | 5 LexA | 23.50 | T3-1 | 5 LexA | | 21.3 | T6-1 | 5 LexA | 25.94 |  |
| T0-1 | | 6 47A-PEPG | 26.03 | T3-1 | 6 47A-PEPG | | 16.2 | T6-1 | 6 47A-PEPG | 19.98 |  |
| T0-1 | | 6 47A-PEPG | 25.66 | T3-1 | 6 47A-PEPG | | 15.6 | T6-1 | 6 47A-PEPG | 19.35 |  |
| T0-1 | | 6 47A-PEPG | 25.16 | T3-1 | 6 47A-PEPG | | 15.2 | T6-1 | 6 47A-PEPG | 18.93 |  |
| T0-1 | | 7 47A-XRE | 25.75 | T3-1 | 7 47A-XRE | | 19.2 | T6-1 | 7 47A-XRE | 23.47 |  |
| T0-1 | | 7 47A-XRE | 25.03 | T3-1 | 7 47A-XRE | | 18.5 | T6-1 | 7 47A-XRE | 22.94 |  |
| T0-1 | | 7 47A-XRE | 25.32 | T3-1 | 7 47A-XRE | | 19.1 | T6-1 | 7 47A-XRE | 23.37 |  |
| T0-1 | | 8 47A-EXO | 23.69 | T3-1 | 8 47A-EXO | | 16.5 | T6-1 | 8 47A-EXO | 20.94 |  |
| T0-1 | | 8 47A-EXO | 22.69 | T3-1 | 8 47A-EXO | | 15.9 | T6-1 | 8 47A-EXO | 20.34 |  |
| T0-1 | | 8 47A-EXO | 22.72 | T3-1 | 8 47A-EXO | | 15.7 | T6-1 | 8 47A-EXO | 20.24 |  |
| T0-1 | | 9 Pro PEPG | 23.05 | T3-1 | 9 Pro PEPG | | 19 | T6-1 | 9 Pro PEPG | 22.66 |  |
| T0-1 | | 9 Pro PEPG | 22.71 | T3-1 | 9 Pro PEPG | | 18.6 | T6-1 | 9 Pro PEPG | 22.06 |  |
| T0-1 | | 9 Pro PEPG | 22.36 | T3-1 | 9 Pro PEPG | | 18.1 | T6-1 | 9 Pro PEPG | 21.85 |  |
| T0-1 | | 10 Pro Rad | 25.41 | T3-1 | 10 Pro Rad | | 19.8 | T6-1 | 10 Pro Rad | 23.75 |  |
| T0-1 | | 10 Pro Rad | 24.59 | T3-1 | 10 Pro Rad | | 18.9 | T6-1 | 10 Pro Rad | 22.89 |  |
| T0-1 | | 10 Pro Rad | 24.83 | T3-1 | 10 Pro Rad | | 19.1 | T6-1 | 10 Pro Rad | 23.14 |  |
| T0-1 | | 11 Pro SSB | 25.19 | T3-1 | 11 Pro SSB | | 18.7 | T6-1 | 11 Pro SSB | 23.07 |  |
| T0-1 | | 11 Pro SSB | 24.72 | T3-1 | 11 Pro SSB | | 18.3 | T6-1 | 11 Pro SSB | 22.51 |  |
| T0-1 | | 11 Pro SSB | 24.34 | T3-1 | 11 Pro SSB | | 18.2 | T6-1 | 11 Pro SSB | 22.28 |  |
| T0-2 | | 1 map | 21.60 | T3-2 | 1 map | | 21.9 | T6-2 | 1 map | 26.64 |  |
| T0-2 | | 1 map | 21.17 | T3-2 | 1 map | | 21.7 | T6-2 | 1 map | 26.71 |  |
| T0-2 | | 1 map | 22.20 | T3-2 | 1 map | | 22.3 | T6-2 | 1 map | 27.28 |  |
| T0-2 | | 2 alaS | 22.69 | T3-2 | 2 alaS | | 22.8 | T6-2 | 2 alaS | 27.27 |  |
| T0-2 | | 2 alaS | 22.74 | T3-2 | 2 alaS | | 22.9 | T6-2 | 2 alaS | 26.95 |  |
| T0-2 | | 2 alaS | 22.92 | T3-2 | 2 alaS | | 23 | T6-2 | 2 alaS | 27.11 |  |
| T0-2 | | 3 rpoC | 17.13 | T3-2 | 3 rpoC | | 19.7 | T6-2 | 3 rpoC | 23.38 |  |
| T0-2 | | 3 rpoC | 16.78 | T3-2 | 3 rpoC | | 19.4 | T6-2 | 3 rpoC | 22.92 |  |
| T0-2 | | 3 rpoC | 16.97 | T3-2 | 3 rpoC | | 19.7 | T6-2 | 3 rpoC | 23.37 |  |
| T0-2 | | 4 RecA | 21.00 | T3-2 | 4 RecA | | 19.9 | T6-2 | 4 RecA | 24.22 |  |
| T0-2 | | 4 RecA | 20.72 | T3-2 | 4 RecA | | 19.6 | T6-2 | 4 RecA | 24.21 |  |
| T0-2 | | 4 RecA | 21.20 | T3-2 | 4 RecA | | 20.1 | T6-2 | 4 RecA | 24.6 |  |
| T0-2 | | 5 LexA | 23.47 | T3-2 | 5 LexA | | 21.1 | T6-2 | 5 LexA | 25.33 |  |
| T0-2 | | 5 LexA | 23.81 | T3-2 | 5 LexA | | 21.4 | T6-2 | 5 LexA | 25.71 |  |
| T0-2 | | 5 LexA | 23.48 | T3-2 | 5 LexA | | 21.4 | T6-2 | 5 LexA | 25.13 |  |
| T0-2 | | 6 47A-PEPG | 26.06 | T3-2 | 6 47A-PEPG | | 15 | T6-2 | 6 47A-PEPG | 18.39 |  |
| T0-2 | | 6 47A-PEPG | 25.86 | T3-2 | 6 47A-PEPG | | 15 | T6-2 | 6 47A-PEPG | 18.35 |  |
| T0-2 | | 6 47A-PEPG | 26.05 | T3-2 | 6 47A-PEPG | | 15 | T6-2 | 6 47A-PEPG | 18.22 |  |
| T0-2 | | 7 47A-XRE | 24.01 | T3-2 | 7 47A-XRE | | 18.7 | T6-2 | 7 47A-XRE | 22.8 |  |
| T0-2 | | 7 47A-XRE | 24.24 | T3-2 | 7 47A-XRE | | 18.9 | T6-2 | 7 47A-XRE | 22.85 |  |
| T0-2 | | 7 47A-XRE | 24.48 | T3-2 | 7 47A-XRE | | 19.2 | T6-2 | 7 47A-XRE | 23.05 |  |
| T0-2 | | 8 47A-EXO | 22.53 | T3-2 | 8 47A-EXO | | 15.5 | T6-2 | 8 47A-EXO | 19.46 |  |
| T0-2 | | 8 47A-EXO | 22.45 | T3-2 | 8 47A-EXO | | 15.3 | T6-2 | 8 47A-EXO | 19.39 |  |
| T0-2 | | 8 47A-EXO | 22.67 | T3-2 | 8 47A-EXO | | 15.5 | T6-2 | 8 47A-EXO | 19.54 |  |
| T0-2 | | 9 Pro PEPG | 22.82 | T3-2 | 9 Pro PEPG | | 18.7 | T6-2 | 9 Pro PEPG | 21.75 |  |
| T0-2 | | 9 Pro PEPG | 22.95 | T3-2 | 9 Pro PEPG | | 18.9 | T6-2 | 9 Pro PEPG | 21.97 |  |
| T0-2 | | 9 Pro PEPG | 22.95 | T3-2 | 9 Pro PEPG | | 18.7 | T6-2 | 9 Pro PEPG | 21.82 |  |
| T0-2 | | 10 Pro Rad | 24.85 | T3-2 | 10 Pro Rad | | 18.7 | T6-2 | 10 Pro Rad | 22.79 |  |
| T0-2 | | 10 Pro Rad | 24.09 | T3-2 | 10 Pro Rad | | 18.5 | T6-2 | 10 Pro Rad | 21.92 |  |
| T0-2 | | 10 Pro Rad | 24.95 | T3-2 | 10 Pro Rad | | 19.2 | T6-2 | 10 Pro Rad | 22.68 |  |
| T0-2 | | 11 Pro SSB | 24.79 | T3-2 | 11 Pro SSB | | 18.6 | T6-2 | 11 Pro SSB | 22.18 |  |
| T0-2 | | 11 Pro SSB | 24.56 | T3-2 | 11 Pro SSB | | 18.2 | T6-2 | 11 Pro SSB | 21.86 |  |
| T0-2 | | 11 Pro SSB | 24.68 | T3-2 | 11 Pro SSB | | 18.5 | T6-2 | 11 Pro SSB | 22.08 |  |
| T0-3 | | 1 map | 22.82 | T3-3 | 1 map | | 23 | T6-3 | 1 map | 27.52 |  |
| T0-3 | | 1 map | 23.34 | T3-3 | 1 map | | 23.3 | T6-3 | 1 map | 27.9 |  |
| T0-3 | | 1map | 23.08 | T3-3 | 1 map | | 23.14 | T6-3 | 1 map | 27.71 |  |
| T0-3 | | 2 alaS | 23.35 | T3-3 | 2 alaS | | 23.2 | T6-3 | 2 alaS | 27.93 |  |
| T0-3 | | 2 alaS | 23.74 | T3-3 | 2 alaS | | 23.8 | T6-3 | 2 alaS | 27.72 |  |
| T0-3 | | 2 alaS | 23.55 | T3-3 | 2 alaS | | 23.52 | T6-3 | 2 alaS | 27.83 |  |
| T0-3 | | 3 rpoC | 17.89 | T3-3 | 3 rpoC | | 20.5 | T6-3 | 3 rpoC | 23.9 |  |
| T0-3 | | 3 rpoC | 18.05 | T3-3 | 3 rpoC | | 20.7 | T6-3 | 3 rpoC | 24.1 |  |
| T0-3 | | 3 rpoC | 17.97 | T3-3 | 3 rpoC | | 20.59 | T6-3 | 3 rpoC | 24.00 |  |
| T0-3 | | 4 RecA | 23.08 | T3-3 | 4 RecA | | 21.4 | T6-3 | 4 RecA | 25.4 |  |
| T0-3 | | 4 RecA | 21.23 | T3-3 | 4 RecA | | 20.2 | T6-3 | 4 RecA | 24.52 |  |
| T0-3 | | 4 RecA | 22.03 | T3-3 | 4 RecA | | 21.3 | T6-3 | 4 RecA | 25.13 |  |
| T0-3 | | 5 LexA | 24.90 | T3-3 | 5 LexA | | 21.9 | T6-3 | 5 LexA | 25.99 |  |
| T0-3 | | 5 LexA | 23.99 | T3-3 | 5 LexA | | 21.4 | T6-3 | 5 LexA | 25.61 |  |
| T0-3 | | 5 LexA | 24.83 | T3-3 | 5 LexA | | 21.8 | T6-3 | 5 LexA | 26.82 |  |
| T0-3 | | 6 47A-PEPG | 27.53 | T3-3 | 6 47A-PEPG | | 16.1 | T6-3 | 6 47A-PEPG | 18.77 |  |
| T0-3 | | 6 47A-PEPG | 27.04 | T3-3 | 6 47A-PEPG | | 15.6 | T6-3 | 6 47A-PEPG | 18.63 |  |
| T0-3 | | 6 47A-PEPG | 27.62 | T3-3 | 6 47A-PEPG | | 16.2 | T6-3 | 6 47A-PEPG | 19.05 |  |
| T0-3 | | 7 47A-XRE | 23.70 | T3-3 | 7 47A-XRE | | 18.2 | T6-3 | 7 47A-XRE | 22.08 |  |
| T0-3 | | 7 47A-XRE | 24.90 | T3-3 | 7 47A-XRE | | 19.4 | T6-3 | 7 47A-XRE | 23.49 |  |
| T0-3 | | 7 47A-XRE | 25.38 | T3-3 | 7 47A-XRE | | 19.9 | T6-3 | 7 47A-XRE | 23.62 |  |
| T0-3 | | 8 47A-EXO | 23.37 | T3-3 | 8 47A-EXO | | 15.8 | T6-3 | 8 47A-EXO | 19.7 |  |
| T0-3 | | 8 47A-EXO | 23.36 | T3-3 | 8 47A-EXO | | 16 | T6-3 | 8 47A-EXO | 19.89 |  |
| T0-3 | | 8 47A-EXO | 24.51 | T3-3 | 8 47A-EXO | | 17.2 | T6-3 | 8 47A-EXO | 20.77 |  |
| T0-3 | | 9 Pro PEPG | 23.47 | T3-3 | 9 Pro PEPG | | 19 | T6-3 | 9 Pro PEPG | 22.11 |  |
| T0-3 | | 9 Pro PEPG | 23.76 | T3-3 | 9 Pro PEPG | | 19.5 | T6-3 | 9 Pro PEPG | 22.29 |  |
| T0-3 | | 9 Pro PEPG | 23.23 | T3-3 | 9 Pro PEPG | | 18.8 | T6-3 | 9 Pro PEPG | 21.78 |  |
| T0-3 | | 10 Pro Rad | 25.61 | T3-3 | 10 Pro Rad | | 19.5 | T6-3 | 10 Pro Rad | 22.92 |  |
| T0-3 | | 10 Pro Rad | 26.16 | T3-3 | 10 Pro Rad | | 20.1 | T6-3 | 10 Pro Rad | 23.54 |  |
| T0-3 | | 10 Pro Rad | 26.11 | T3-3 | 10 Pro Rad | | 20.2 | T6-3 | 10 Pro Rad | 23.37 |  |
| T0-3 | | 11 Pro SSB | 25.10 | T3-3 | 11 Pro SSB | | 18.8 | T6-3 | 11 Pro SSB | 22.3 |  |
| T0-3 | | 11 Pro SSB | 26.09 | T3-3 | 11 Pro SSB | | 19.5 | T6-3 | 11 Pro SSB | 23.17 |  |
| T0-3 | | 11 Pro SSB | 26.30 | T3-3 | 11 Pro SSB | | 19.8 | T6-3 | 11 Pro SSB | 23.57 |  |
| **Non-infected controls** | | | |  |  |  | |  |  |  | |
| time point (-rep) | gene | | C(t) | time point (-rep) | gene | C(t) | | time point  (-rep) | gene | C(t) | |
| T0-1 | 1 map | | 26.34 | T3-1 | 1 map | 25.10 | | T6-1 | 1 map | 25.25 | |
| T0-1 | 1 map | | 26.09 | T3-1 | 1 map | 24.98 | | T6-1 | 1 map | 25.05 | |
| T0-1 | 1 map | | 26.03 | T3-1 | 1 map | 24.80 | | T6-1 | 1 map | 24.95 | |
| T0-1 | 2 alaS | | 26.34 | T3-1 | 2 alaS | 26.23 | | T6-1 | 2 alaS | 25.05 | |
| T0-1 | 2 alaS | | 25.72 | T3-1 | 2 alaS | 26.19 | | T6-1 | 2 alaS | 24.58 | |
| T0-1 | 2 alaS | | 25.52 | T3-1 | 2 alaS | 26.19 | | T6-1 | 2 alaS | 24.46 | |
| T0-1 | 3 rpoC | | 21.50 | T3-1 | 3 rpoC | 21.79 | | T6-1 | 3 rpoC | 20.31 | |
| T0-1 | 3 rpoC | | 21.17 | T3-1 | 3 rpoC | 21.59 | | T6-1 | 3 rpoC | 20.10 | |
| T0-1 | 3 rpoC | | 21.34 | T3-1 | 3 rpoC | 21.69 | | T6-1 | 3 rpoC | 20.08 | |
| T0-1 | 4 RecA | | 25.32 | T3-1 | 4 RecA | 23.38 | | T6-1 | 4 RecA | 23.55 | |
| T0-1 | 4 RecA | | 24.78 | T3-1 | 4 RecA | 23.19 | | T6-1 | 4 RecA | 22.96 | |
| T0-1 | 4 RecA | | 24.83 | T3-1 | 4 RecA | 23.23 | | T6-1 | 4 RecA | 23.20 | |
| T0-1 | 5 LexA | | 26.91 | T3-1 | 5 LexA | 26.92 | | T6-1 | 5 LexA | 25.17 | |
| T0-1 | 5 LexA | | 26.27 | T3-1 | 5 LexA | 26.29 | | T6-1 | 5 LexA | 24.80 | |
| T0-1 | 5 LexA | | 25.84 | T3-1 | 5 LexA | 25.45 | | T6-1 | 5 LexA | 24.12 | |
| T0-1 | 8 47A-EXO | | 25.76 | T3-1 | 8 47A-EXO | 25.89 | | T6-1 | 8 47A-EXO | 20.95 | |
| T0-1 | 8 47A-EXO | | 25.00 | T3-1 | 8 47A-EXO | 24.92 | | T6-1 | 8 47A-EXO | 20.66 | |
| T0-1 | 8 47A-EXO | | 25.08 | T3-1 | 8 47A-EXO | 25.06 | | T6-1 | 8 47A-EXO | 20.36 | |
| T0-1 | 9 Pro PEPG | | 25.01 | T3-1 | 9 Pro PEPG | 25.01 | | T6-1 | 9 Pro PEPG | 21.49 | |
| T0-1 | 9 Pro PEPG | | 24.51 | T3-1 | 9 Pro PEPG | 24.48 | | T6-1 | 9 Pro PEPG | 21.10 | |
| T0-1 | 9 Pro PEPG | | 24.32 | T3-1 | 9 Pro PEPG | 24.13 | | T6-1 | 9 Pro PEPG | 20.47 | |
| T0-1 | 10 Pro Rad | | 27.14 | T3-1 | 10 Pro Rad | 27.84 | | T6-1 | 10 Pro Rad | 24.24 | |
| T0-1 | 10 Pro Rad | | 26.26 | T3-1 | 10 Pro Rad | 26.99 | | T6-1 | 10 Pro Rad | 23.79 | |
| T0-1 | 10 Pro Rad | | 26.39 | T3-1 | 10 Pro Rad | 27.57 | | T6-1 | 10 Pro Rad | 24.23 | |
| T0-1 | 11 Pro SSB | | 26.71 | T3-1 | 11 Pro SSB | 28.06 | | T6-1 | 11 Pro SSB | 23.88 | |
| T0-1 | 11 Pro SSB | | 25.85 | T3-1 | 11 Pro SSB | 27.11 | | T6-1 | 11 Pro SSB | 23.32 | |
| T0-1 | 11 Pro SSB | | 25.67 | T3-1 | 11 Pro SSB | 26.58 | | T6-1 | 11 Pro SSB | 23.29 | |
| T0-2 | 1 map | | 25.76 | T3-2 | 1 map | 23.49 | | T6-2 | 1 map | 23.61 | |
| T0-2 | 1 map | | 25.49 | T3-2 | 1 map | 23.02 | | T6-2 | 1 map | 23.39 | |
| T0-2 | 1 map | | 26.45 | T3-2 | 1 map | 24.11 | | T6-2 | 1 map | 24.48 | |
| T0-2 | 2 alaS | | 26.19 | T3-2 | 2 alaS | 24.14 | | T6-2 | 2 alaS | 23.69 | |
| T0-2 | 2 alaS | | 25.88 | T3-2 | 2 alaS | 24.24 | | T6-2 | 2 alaS | 23.99 | |
| T0-2 | 2 alaS | | 26.04 | T3-2 | 2 alaS | 24.19 | | T6-2 | 2 alaS | 23.92 | |
| T0-2 | 3 rpoC | | 21.00 | T3-2 | 3 rpoC | 20.61 | | T6-2 | 3 rpoC | 19.58 | |
| T0-2 | 3 rpoC | | 20.98 | T3-2 | 3 rpoC | 20.19 | | T6-2 | 3 rpoC | 19.44 | |
| T0-2 | 3 rpoC | | 21.13 | T3-2 | 3 rpoC | 20.45 | | T6-2 | 3 rpoC | 19.61 | |
| T0-2 | 4 RecA | | 24.82 | T3-2 | 4 RecA | 21.95 | | T6-2 | 4 RecA | 22.22 | |
| T0-2 | 4 RecA | | 24.79 | T3-2 | 4 RecA | 21.64 | | T6-2 | 4 RecA | 23.25 | |
| T0-2 | 4 RecA | | 25.13 | T3-2 | 4 RecA | 22.22 | | T6-2 | 4 RecA | 22.58 | |
| T0-2 | 5 LexA | | 26.12 | T3-2 | 5 LexA | 24.49 | | T6-2 | 5 LexA | 23.50 | |
| T0-2 | 5 LexA | | 26.24 | T3-2 | 5 LexA | 25.02 | | T6-2 | 5 LexA | 23.66 | |
| T0-2 | 5 LexA | | 26.80 | T3-2 | 5 LexA | 24.92 | | T6-2 | 5 LexA | 23.64 | |
| T0-2 | 8 47A-EXO | | 25.33 | T3-2 | 8 47A-EXO | 23.99 | | T6-2 | 8 47A-EXO | 20.98 | |
| T0-2 | 8 47A-EXO | | 25.15 | T3-2 | 8 47A-EXO | 23.70 | | T6-2 | 8 47A-EXO | 21.02 | |
| T0-2 | 8 47A-EXO | | 25.33 | T3-2 | 8 47A-EXO | 23.79 | | T6-2 | 8 47A-EXO | 21.00 | |
| T0-2 | 9 Pro PEPG | | 25.26 | T3-2 | 9 Pro PEPG | 23.64 | | T6-2 | 9 Pro PEPG | 20.15 | |
| T0-2 | 9 Pro PEPG | | 25.37 | T3-2 | 9 Pro PEPG | 23.80 | | T6-2 | 9 Pro PEPG | 20.00 | |
| T0-2 | 9 Pro PEPG | | 25.18 | T3-2 | 9 Pro PEPG | 23.79 | | T6-2 | 9 Pro PEPG | 20.23 | |
| T0-2 | 10 Pro Rad | | 27.00 | T3-2 | 10 Pro Rad | 26.63 | | T6-2 | 10 Pro Rad | 23.83 | |
| T0-2 | 10 Pro Rad | | 26.28 | T3-2 | 10 Pro Rad | 26.02 | | T6-2 | 10 Pro Rad | 24.37 | |
| T0-2 | 10 Pro Rad | | 27.61 | T3-2 | 10 Pro Rad | 27.03 | | T6-2 | 10 Pro Rad | 24.56 | |
| T0-2 | 11 Pro SSB | | 26.74 | T3-2 | 11 Pro SSB | 26.53 | | T6-2 | 11 Pro SSB | 23.79 | |
| T0-2 | 11 Pro SSB | | 26.41 | T3-2 | 11 Pro SSB | 26.46 | | T6-2 | 11 Pro SSB | 23.62 | |
| T0-2 | 11 Pro SSB | | 26.94 | T3-2 | 11 Pro SSB | 26.73 | | T6-2 | 11 Pro SSB | 23.67 | |
| T0-3 | 1 map | | 23.66 | T3-3 | 1 map | 24.62 | | T6-3 | 1 map | 24.20 | |
| T0-3 | 1 map | | 24.36 | T3-3 | 1 map | 24.77 | | T6-3 | 1 map | 24.62 | |
| T0-3 | 1 map | | 24.01 | T3-3 | 1 map | 24.70 | | T6-3 | 1 map | 24.17 | |
| T0-3 | 2 alaS | | 24.28 | T3-3 | 2 alaS | 24.68 | | T6-3 | 2 alaS | 24.25 | |
| T0-3 | 2 alaS | | 24.60 | T3-3 | 2 alaS | 24.98 | | T6-3 | 2 alaS | 24.86 | |
| T0-3 | 2 alaS | | 24.97 | T3-3 | 2 alaS | 25.20 | | T6-3 | 2 alaS | 23.88 | |
| T0-3 | 3 rpoC | | 19.06 | T3-3 | 3 rpoC | 21.02 | | T6-3 | 3 rpoC | 20.12 | |
| T0-3 | 3 rpoC | | 19.42 | T3-3 | 3 rpoC | 21.27 | | T6-3 | 3 rpoC | 20.18 | |
| T0-3 | 3 rpoC | | 19.52 | T3-3 | 3 rpoC | 21.50 | | T6-3 | 3 rpoC | 19.97 | |
| T0-3 | 4 RecA | | 23.38 | T3-3 | 4 RecA | 23.48 | | T6-3 | 4 RecA | 22.14 | |
| T0-3 | 4 RecA | | 22.19 | T3-3 | 4 RecA | 22.53 | | T6-3 | 4 RecA | 22.75 | |
| T0-3 | 4 RecA | | 22.93 | T3-3 | 4 RecA | 22.88 | | T6-3 | 4 RecA | 24.45 | |
| T0-3 | 5 LexA | | 24.98 | T3-3 | 5 LexA | 25.84 | | T6-3 | 5 LexA | 23.97 | |
| T0-3 | 5 LexA | | 24.52 | T3-3 | 5 LexA | 25.40 | | T6-3 | 5 LexA | 25.16 | |
| T0-3 | 5 LexA | | 25.82 | T3-3 | 5 LexA | 26.12 | | T6-3 | 5 LexA | 21.39 | |
| T0-3 | 8 47A-EXO | | 23.76 | T3-3 | 8 47A-EXO | 24.30 | | T6-3 | 8 47A-EXO | 21.74 | |
| T0-3 | 8 47A-EXO | | 23.90 | T3-3 | 8 47A-EXO | 24.46 | | T6-3 | 8 47A-EXO | 21.70 | |
| T0-3 | 8 47A-EXO | | 25.33 | T3-3 | 8 47A-EXO | 25.58 | | T6-3 | 8 47A-EXO | 22.07 | |
| T0-3 | 9 Pro PEPG | | 23.30 | T3-3 | 9 Pro PEPG | 23.81 | | T6-3 | 9 Pro PEPG | 20.84 | |
| T0-3 | 9 Pro PEPG | | 23.70 | T3-3 | 9 Pro PEPG | 24.18 | | T6-3 | 9 Pro PEPG | 21.59 | |
| T0-3 | 9 Pro PEPG | | 23.98 | T3-3 | 9 Pro PEPG | 24.52 | | T6-3 | 9 Pro PEPG | 25.34 | |
| T0-3 | 10 Pro Rad | | 26.44 | T3-3 | 10 Pro Rad | 27.21 | | T6-3 | 10 Pro Rad | 25.44 | |
| T0-3 | 10 Pro Rad | | 27.15 | T3-3 | 10 Pro Rad | 27.82 | | T6-3 | 10 Pro Rad | 25.91 | |
| T0-3 | 10 Pro Rad | | 26.92 | T3-3 | 10 Pro Rad | 27.95 | | T6-3 | 10 Pro Rad | 25.64 | |
| T0-3 | 11 Pro SSB | | 26.06 | T3-3 | 11 Pro SSB | 26.83 | | T6-3 | 11 Pro SSB | 24.60 | |
| T0-3 | 11 Pro SSB | | 26.82 | T3-3 | 11 Pro SSB | 27.89 | | T6-3 | 11 Pro SSB | 25.97 | |
| T0-3 | 11 Pro SSB | | 26.91 | T3-3 | 11 Pro SSB | 27.85 | | T6-3 | 11 Pro SSB | 22.01 | |
|  |  | |  |  |  |  | | No RT | SBG1 | 29.88 | |
|  |  | |  |  |  |  | | No RT | SBG1 | 28.14 | |
|  |  | |  |  |  |  | | No RT | SBG1 | 28.57 | |
|  |  | |  |  |  |  | | No RT | SBG1 | 28.88 | |
|  |  | |  |  |  |  | | No RT | SBG1 | 29.12 | |
|  |  | |  |  |  |  | | No RT | SBG1 | 28.31 | |
|  |  | |  |  |  |  | | No RT | SBG1 | 28.65 | |
|  |  | |  |  |  |  | | No RT | SBG1 | 29.09 | |

**Table S9. Optical density (OD 540 nm) for Fig 4A**

| **CB-D** | | | | | | **CB-A** | | | | | |
| --- | --- | --- | --- | --- | --- | --- | --- | --- | --- | --- | --- |
| **Time (hours)** | **Rep 1** | **Rep 2** | **Rep 3** | **CB-D av** | **Stdev** | **Time** | **Rep 1** | **Rep 2** | **Rep 3** | **CB-A av** | **Stdev** |
| **-1** | **0.064** | **0.07** | **0.069** | **0.068** | **0.003** | **-1** | **0.063** | **0.065** | **0.077** | **0.068** | **0.008** |
| **-2** | **0.109** | **0.118** | **0.116** | **0.114** | **0.005** | **-2** | **0.105** | **0.106** | **0.117** | **0.109** | **0.007** |
| **0** | **0.173** | **0.187** | **0.179** | **0.180** | **0.007** | **0** | **0.168** | **0.164** | **0.166** | **0.166** | **0.002** |
| **1** | **0.267** | **0.275** | **0.275** | **0.272** | **0.005** | **1** | **0.248** | **0.242** | **0.245** | **0.245** | **0.003** |
| **2** | **0.359** | **0.388** | **0.384** | **0.377** | **0.016** | **2** | **0.325** | **0.325** | **0.319** | **0.323** | **0.003** |
| **3** | **0.465** | **0.512** | **0.506** | **0.494** | **0.026** | **3** | **0.417** | **0.441** | **0.406** | **0.421** | **0.018** |
| **4** | **0.63** | **0.646** | **0.664** | **0.647** | **0.017** | **4** | **0.525** | **0.567** | **0.523** | **0.538** | **0.025** |
| **5** | **0.748** | **0.792** | **0.766** | **0.769** | **0.022** | **5** | **0.628** | **0.659** | **0.615** | **0.634** | **0.023** |
| **6** | **0.852** | **0.893** | **0.866** | **0.870** | **0.021** | **6** | **0.758** | **0.791** | **0.712** | **0.754** | **0.040** |
| **7** | **0.928** | **0.966** | **0.957** | **0.950** | **0.020** | **7** | **0.835** | **0.843** | **0.776** | **0.818** | **0.037** |
| **8** | **1.018** | **1.054** | **1.016** | **1.029** | **0.021** | **8** | **0.9** | **0.914** | **0.848** | **0.887** | **0.035** |
| **27** | **1.426** | **1.445** | **1.46** | **1.444** | **0.017** | **27** | **0.967** | **0.978** | **0.981** | **0.975** | **0.007** |
| **28** | **1.448** | **1.451** | **1.452** | **1.450** | **0.002** | **28** | **1.182** | **0.952** | **0.951** | **1.028** | **0.133** |
| **29** | **1.45** | **1.46** | **1.452** | **1.454** | **0.005** | **29** | **1.228** | **0.941** | **0.935** | **1.035** | **0.167** |
| **30** | **1.448** | **1.454** | **1.432** | **1.445** | **0.011** | **30** | **1.224** | **0.946** | **0.923** | **1.031** | **0.168** |

**Table S10. Optical density (OD 600 nm) for Fig 4B**

|  | **CB-D** | **CB-A** |
| --- | --- | --- |
| **Rep1** | **0.043** | **0.0873** |
|  | **0.0442** | **0.0975** |
|  | **0.0355** | **0.0682** |
| **Rep 2** | **0.0315** | **0.0769** |
|  | **0.0464** | **0.0826** |
|  | **0.0421** | **0.0689** |
| **Rep 3** | **0.0249** | **0.0917** |
|  | **0.049** | **0.0839** |
|  | **0.0481** | **0.0796** |

**Table S11. Optical density (OD 540nm) readings for Fig 5A.**

|  | **CB-A** | **CB-D** | **Co-Culture** |
| --- | --- | --- | --- |
|  | **0.825** | **1.266** | **0.681** |
|  | **0.807** | **1.122** | **0.863** |
|  | **0.888** | **1.068** | **0.722** |
| **Av** | **0.84** | **1.152** | **0.7553** |
| **Stdv** | **0.02456** | **0.05909** | **0.05512** |

**Table S12. qPCR data for Fig 5A and B**

| sample type | biological replicate | gene | C(t) | gene copies |  | gene | C(t) | gene copies |
| --- | --- | --- | --- | --- | --- | --- | --- | --- |
| broth culture cell fraction | A | A phage | 6.85 | 6.71E+07 |  | D phage | 7.89 | 3.70E+07 |
| broth culture cell fraction | A | A phage | 6.87 | 6.60E+07 |  | D phage | 8.15 | 3.10E+07 |
| broth culture cell fraction | A | A phage | 6.69 | 7.55E+07 |  | D phage | 7.92 | 3.64E+07 |
| broth culture cell fraction | B | A phage | 6.72 | 7.37E+07 |  | D phage | 9.34 | 1.37E+07 |
| broth culture cell fraction | B | A phage | 6.82 | 6.86E+07 |  | D phage | 9.09 | 1.63E+07 |
| broth culture cell fraction | B | A phage | 6.62 | 7.97E+07 |  | D phage | 9.03 | 1.70E+07 |
| broth culture cell fraction | C | A phage | 7.10 | 5.54E+07 |  | D phage | 9.43 | 1.29E+07 |
| broth culture cell fraction | C | A phage | 7.29 | 4.83E+07 |  | D phage | 8.94 | 1.81E+07 |
| broth culture cell fraction | C | A phage | 7.63 | 3.74E+07 |  | D phage | 9.24 | 1.48E+07 |
| cell free filtrate | A | A phage | 9.32 | 1.05E+07 |  | D phage | 12.69 | 1.39E+06 |
| cell free filtrate | A | A phage | 9.52 | 9.00E+06 |  | D phage | 12.56 | 1.52E+06 |
| cell free filtrate | A | A phage | 9.90 | 6.81E+06 |  | D phage | 12.61 | 1.47E+06 |
| cell free filtrate | B | A phage | 10.60 | 3.92E+06 |  | D phage | 12.95 | 1.16E+06 |
| cell free filtrate | B | A phage | 10.30 | 5.18E+06 |  | D phage | 12.87 | 1.23E+06 |
| cell free filtrate | B | A phage | 10.48 | 4.39E+06 |  | D phage | 13.05 | 1.09E+06 |
| cell free filtrate | C | A phage | 9.81 | 7.25E+06 |  | D phage | 13.47 | 8.18E+05 |
| cell free filtrate | C | A phage | 9.77 | 7.50E+06 |  | D phage | 13.03 | 1.10E+06 |
| cell free filtrate | C | A phage | 9.81 | 7.24E+06 |  | D phage | 13.42 | 8.47E+05 |
|  | Standard | A phage | 27.08 | 10 |  | D phage | 28.86 | 10 |
|  | Standard | A phage | 28.00 | 10 |  | D phage | 28.86 | 10 |
|  | Standard | A phage | 24.82 | 100 |  | D phage | 26.66 | 100 |
|  | Standard | A phage | 24.10 | 100 |  | D phage | 27.34 | 100 |
|  | Standard | A phage | 21.68 | 1000 |  | D phage | 23.47 | 1000 |
|  | Standard | A phage | 22.30 | 1000 |  | D phage | 23.67 | 1000 |
|  | Standard | A phage | 18.70 | 1.00E+04 |  | D phage | 20.24 | 1.00E+04 |
|  | Standard | A phage | 18.40 | 1.00E+04 |  | D phage | 20.47 | 1.00E+04 |
|  | Standard | A phage | 16.10 | 1.00E+05 |  | D phage | 17.11 | 1.00E+05 |
|  | Standard | A phage | 15.70 | 1.00E+05 |  | D phage | 17.15 | 1.00E+05 |
|  | Standard | A phage | 12.40 | 1.00E+06 |  | D phage | 13.21 | 1.00E+06 |
|  | Standard | A phage | 12.60 | 1.00E+06 |  | D phage | 13.51 | 1.00E+06 |
|  | Standard | A phage | 9.41 | 1.00E+07 |  | D phage | 9.48 | 1.00E+07 |
|  | Standard | A phage | 9.64 | 1.00E+07 |  | D phage | 9.55 | 1.00E+07 |
|  | Standard | A phage | 5.58 | 1.00E+08 |  | D phage | 6.04 | 1.00E+08 |
|  | Standard | A phage | 6.33 | 1.00E+08 |  | D phage | 5.96 | 1.00E+08 |
|  | Blank | A phage | 30.50 | 1.33 |  | D phage | 28.70 | 24.48 |
|  | Blank | A phage | 30.20 | 1.683 |  | D phage | 28.81 | 22.67 |
|  | Blank | A phage | 29.30 | 3.352 |  | D phage | 28.13 | 36.22 |
|  |  |  |  |  |  |  |  |  |
| biofilm | A | A phage | 6.58 |  |  | D phage | 5.65 |  |
| biofilm | A | A phage | 5.41 |  |  | D phage | 5.78 |  |
| biofilm | A | A phage | 6.34 |  |  | D phage | 5.83 |  |
| biofilm | B | A phage | 4.77 |  |  | D phage | 4.27 |  |
| biofilm | B | A phage | 4.99 |  |  | D phage | 4.23 |  |
| biofilm | B | A phage | 4.61 |  |  | D phage | 5.38 |  |
| biofilm | C | A phage | 4.71 |  |  | D phage | 3.11 |  |
| biofilm | C | A phage | 4.59 |  |  | D phage | 2.95 |  |
| biofilm | C | A phage | 4.36 |  |  | D phage | 4.09 |  |

**Table S13. Biofilm Crystal Violet assay optical density (OD 600nm) readings for Fig 5B.**

| **48hr biofilm assay** | | | | | | | | | | | | |
| --- | --- | --- | --- | --- | --- | --- | --- | --- | --- | --- | --- | --- |
| **rep** | **Neg-1** | **Neg-2** | **Neg-3** | **CB-D-1** | **CB-D-2** | **CB-D-3** | **CB-A-1** | **CB-A-2** | **CB-A-3** | **Comp-1** | **Comp-2** | **Comp-3** |
| **A** | **0.185** | **0.188** | **0.193** | **0.317** | **0.359** | **0.326** | **0.395** | **0.384** | **0.398** | **0.494** | **0.486** | **0.493** |
| **B** | **0.18** | **0.18** | **0.179** | **0.299** | **0.33** | **0.288** | **0.357** | **0.349** | **0.365** | **0.466** | **0.429** | **0.442** |
| **C** | **0.186** | **0.19** | **0.193** | **0.313** | **0.337** | **0.313** | **0.405** | **0.382** | **0.379** | **0.493** | **0.441** | **0.434** |
| **D** | **0.183** | **0.182** | **0.187** | **0.3** | **0.349** | **0.311** | **0.41** | **0.377** | **0.386** | **0.464** | **0.442** | **0.434** |
| **E** | **0.194** | **0.194** | **0.205** | **0.332** | **0.397** | **0.348** | **0.429** | **0.424** | **0.425** | **0.500** | **0.455** | **0.441** |
| **F** | **0.194** | **0.188** | **0.19** | **0.336** | **0.374** | **0.329** | **0.404** | **0.419** | **0.363** | **0.468** | **0.416** | **0.453** |
| **G** | **0.202** | **0.201** | **0.214** | **0.329** | **0.403** | **0.356** | **0.433** | **0.426** | **0.417** | **0.503** | **0.469** | **0.458** |
| **H** | **0.2** | **0.196** | **0.201** | **0.35** | **0.368** | **0.318** | **0.382** | **0.407** | **0.375** | **0.473** | **0.417** | **0.45** |

**
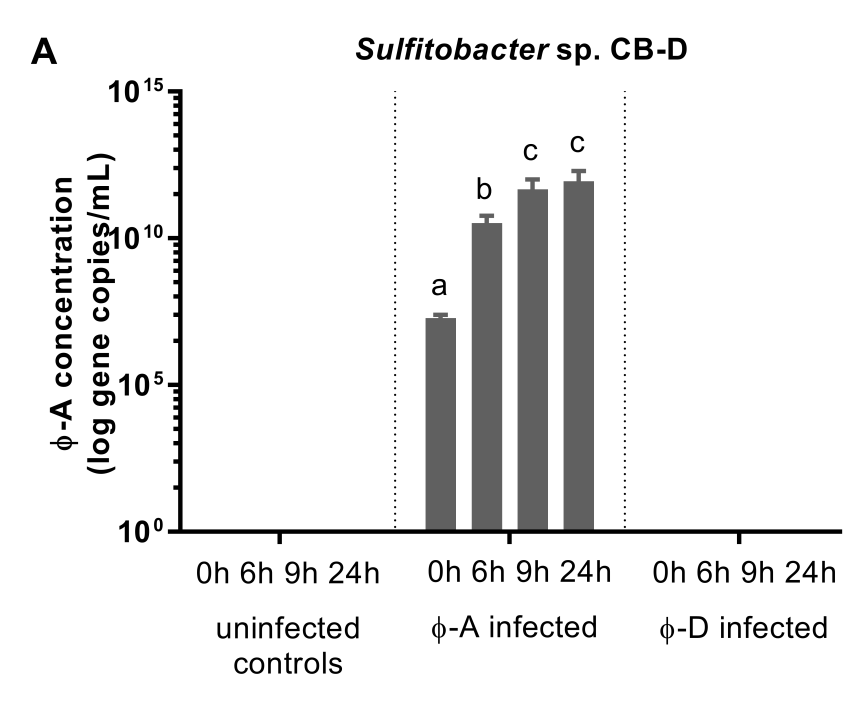

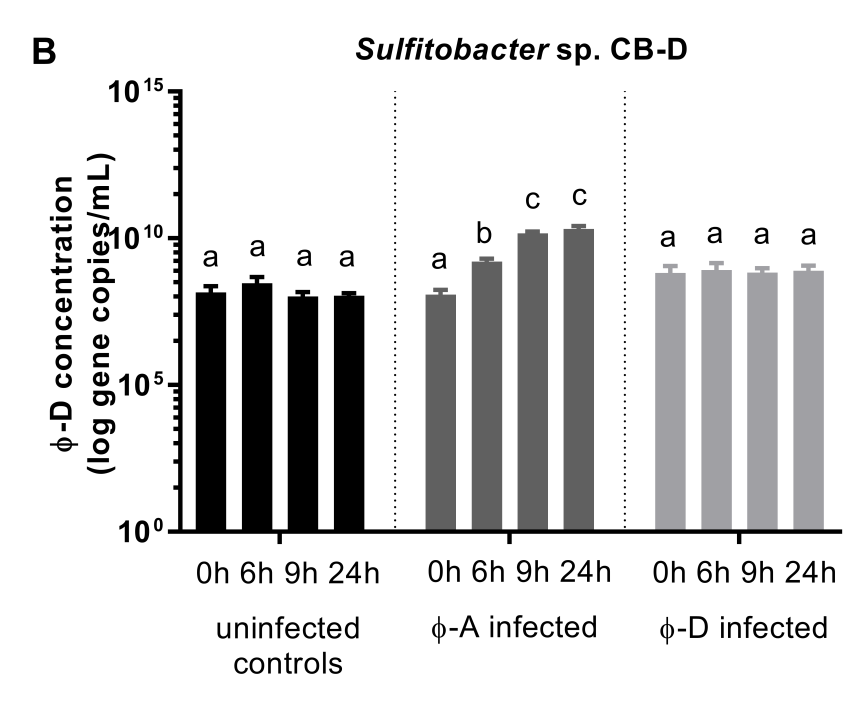

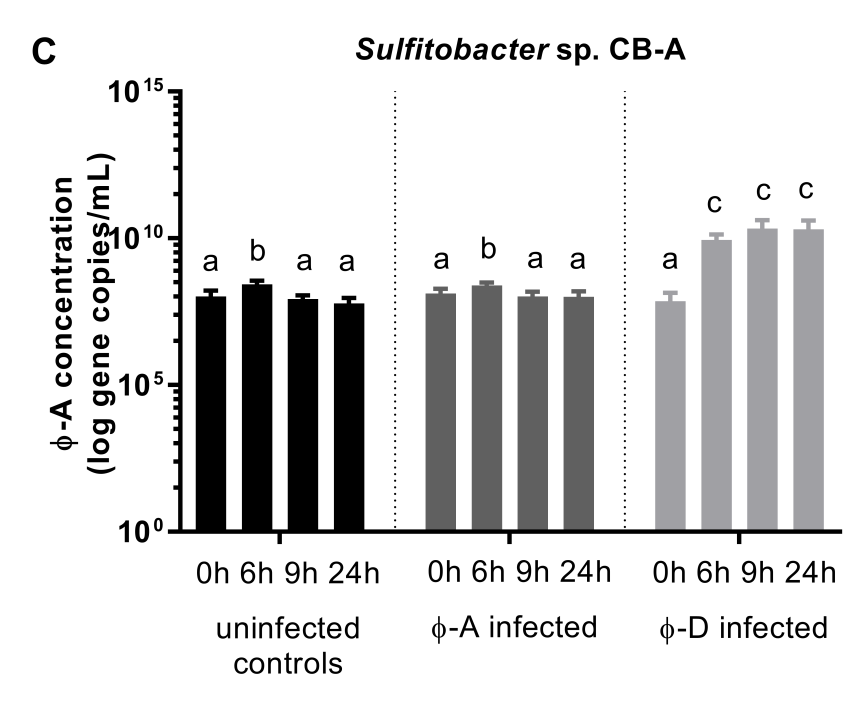

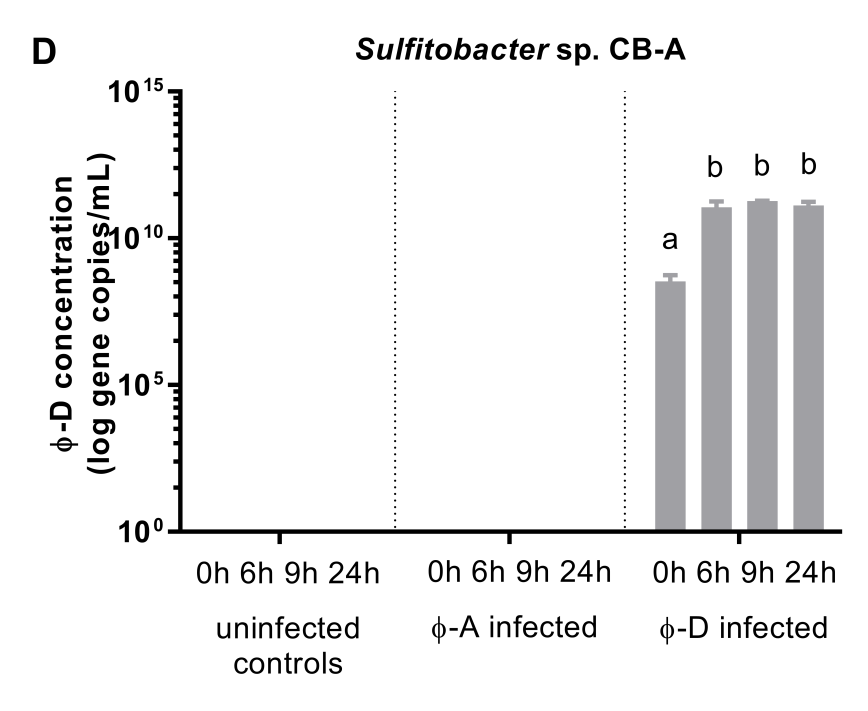
**

**Fig S1**. Quantification of each phage during superinfection resistance tests. **(A)** ɸ-A and **(B)** ɸ-D gene copies during infection of *Sulfitobacter* sp. strain CB-D. **(C)** ɸ-A and **(D)** ɸ-D gene copies during infection of *Sulfitobacter* sp. strain CB-A. Averages of biological triplicates are reported for all treatments. Error bars represent the standard deviation of the mean. Different letters denote columns with significantly different (p<0.05) phage gene copy numbers within a given plot.

**
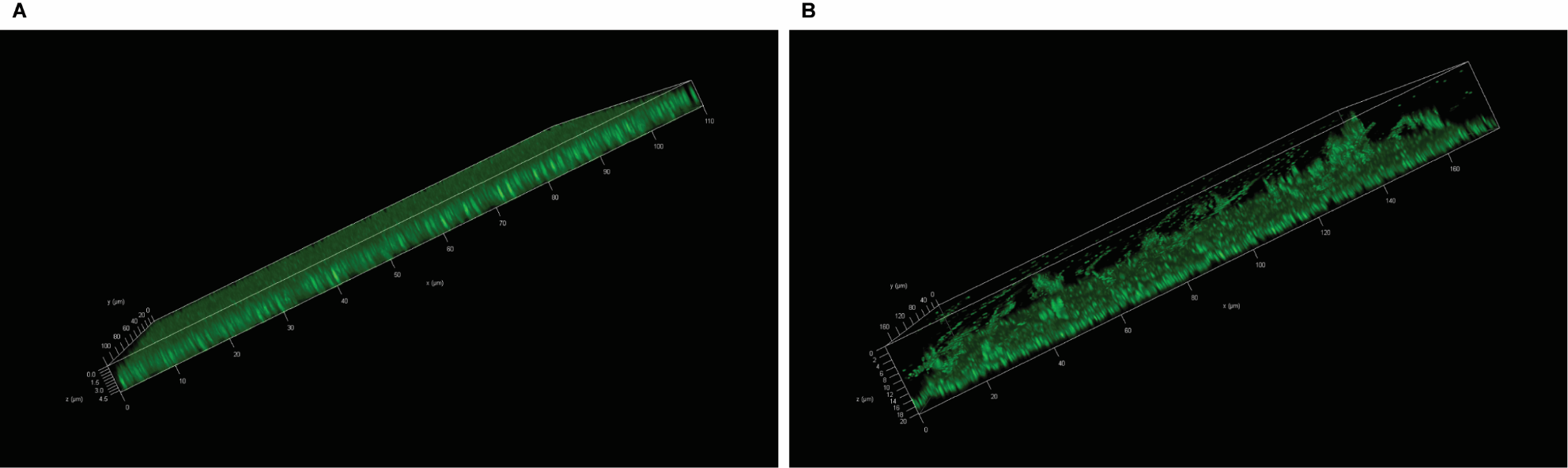
**

**Fig S2**. 3D analysis of **(A)** CB-D and **(B)** CB-A biofilms. Overnight cultures of each strain were sub-cultured, diluted, and grown to early exponential phase (OD_540nm_ = 0.17). 3 mL of each strain was added to FlouroDish glass dishes (World Precision Instruments, Inc.) and incubated without agitation overnight at room temperature. Planktonic bacteria were removed by gentle inversion of the glass dishes and washed twice with standard marine media (SMM). Dishes were left to dry for 15 min, then stained with 3mL 25X SYBR Gold (ThermoFisher Scientific SYBR^TM^ Gold Nucleic Acid Gel Stain) for 30 min. Images were generated using confocal laser microscopy and visualized using a Leica SP8 white light Laser Confocal System. Biofilm reconstruction was performed with the Leica Application Suite X (Las X) software. Confocal microscopy methods were greatly adapted from previously described literature (Townsley and Yildiz 2015) and data were analyzed using Comstat2 (Heydorn et al 2000, Vorregaard 2008). Images were taken from biological triplicate cultures and at least three images were generated per sample.


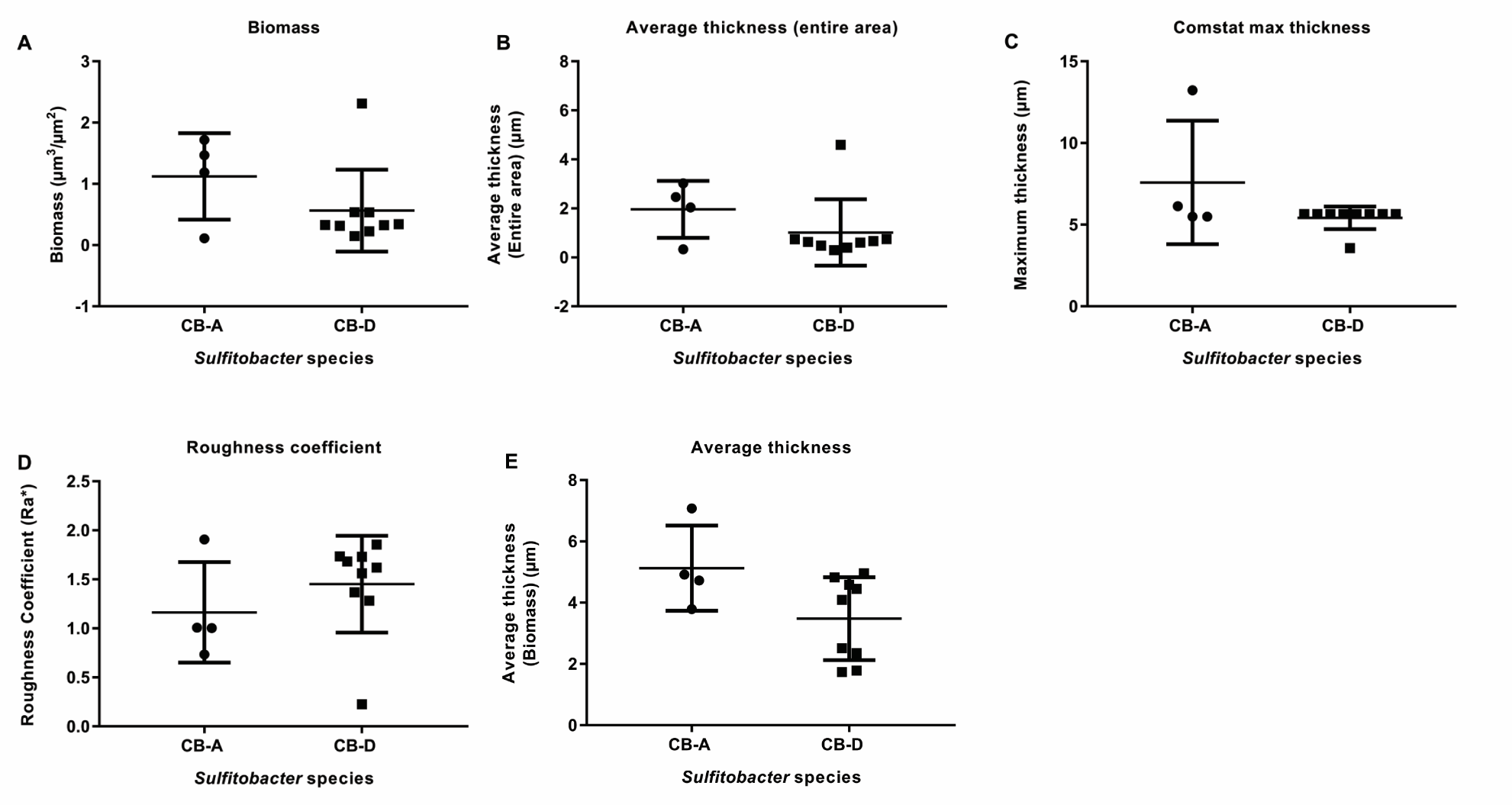


**Fig S3**. Comstat2 analysis of CB-D and CB-A biofilm confocal microscopy data. Biofilm **(A)** biomass, **(B)** average thickness, **(C) C**omstat max thickness, **(D)** roughness coefficient, and **(E)** average thickness. Each data point represents a separate Z stack field of view of the FlouroDish. All parameters showed no significant difference (Student’s t-tests; p<0.05).

ɸ-A

ɸ-D

**Fig S4.** Recruitment of reads from ME-08-09 (~35kb size fraction virome reads) using either ɸ-A or ɸ-D as reference genomes. Read recruitments were done using CLC, default parameters. Single reads mapping in their forward direction are green. Those mapping in reverse direction are red. Non-specific matches are yellow.

**REFERENCES**

Heydorn A, Nielsen AT, Hentzer M, Sternberg C, Givskov M, Ersboll BK *et al* (2000). Quantification of biofilm structures by the novel computer program COMSTAT. *Microbiology-Uk* **146:** 2395-2407.

Townsley L, Yildiz FH (2015). Temperature affects c-di-GMP signalling and biofilm formation in Vibrio cholerae. *Environmental Microbiology* **17:** 4290-4305.

Vorregaard M (2008). Comstat2 - a modern 3D image analysis environment for biofilms, in Informatics and Mathematical Modelling.
